# On the real zeroes of half-integral weight Hecke cusp forms

**DOI:** 10.1007/s00208-026-03393-w

**Published:** 2026-02-18

**Authors:** Jesse Jääsaari

**Affiliations:** Department of Mathematics and Statistics, University of Turku, 20014 Turku, Finland

**Keywords:** Primary 11F37, Secondary 11M06

## Abstract

We examine the distribution of zeroes of half-integral weight Hecke cusp forms on the manifold $\Gamma _0(4)\backslash \mathbb H$ near a cusp at infinity. In analogue of the Ghosh–Sarnak conjecture for classical holomorphic Hecke cusp forms, one expects that almost all of the zeroes sufficiently close to this cusp lie on two vertical geodesics $\text {Re}(s)=-1/2$ and $\text {Re}(s)=0$ as the weight tends to infinity. We show that, for $\gg _\varepsilon K^2/(\log K)^{3/2+\varepsilon }$ of the half-integral weight Hecke cusp forms in the Kohnen plus subspaces with weight bounded by a large parameter *K*, the number of such “real” zeroes grows almost at the expected rate. We also obtain a weaker lower bound for the number of real zeroes that holds for a positive proportion of forms. One of the key ingredients is the estimation of averaged first and second moments of quadratic twists of modular *L*-functions.

## Introduction

Studying the distribution of zeroes of automorphic forms has attracted attention both historically and more recently. A classical result in the theory of holomorphic modular forms for the group $\textrm{SL}_2(\mathbb {Z})$ on the upper half $\mathbb {H}$ of the complex plane is the so-called valence formula. It states that the number of (properly weighted) zeroes of such a form *f* with weight *k* is asymptotically *k*/12 inside the closure of the fundamental domain $\mathcal {D}:=\textrm{SL}_2(\mathbb {Z})\backslash \mathbb H=\{z\in \mathbb {H}{:}\, -1/2\le \text {Re}(z) \le 1/2,\, |z|\ge 1\}$. Interestingly, it turns out that on smaller scales the distribution of zeroes of different types of automorphic forms can vary drastically. For instance, Rankin and Swinnerton-Dyer [45] have shown that all the zeroes of holomorphic Eisenstein series lie on the arc $\{|z|=1\}$ and moreover these zeroes are uniformly distributed there as $k\longrightarrow \infty$. In contrast, powers of the modular discriminant have a single zero of high multiplicity at infinity. Another type of behaviour is displayed by Hecke cusp forms, which form a natural basis for the space of modular forms of a given weight.

For the latter forms Rudnick [46] showed that the equidistribution of zeroes inside $\mathcal {D}$, as the weight of the form tends to infinity, follows from the holomorphic analogue of the Quantum Unique Ergodicity conjecture (QUE) of himself and Sarnak [47], which is spelled out explicitly in [36, 48]. As this conjecture has since been shown to hold by Holowinsky and Soundararajan [16], Rudnick’s equidistribution result is unconditional. Given this, it is natural to wonder about the finer distributional behaviour of the zeroes of Hecke cusp forms, e.g. their distribution within subsets of $\mathcal {D}$ that shrink as the weight grows.

Such questions were first explored by Ghosh and Sarnak [12] who considered the distribution of zeroes in shrinking domains around the cusp of the modular surface $\mathcal {D}$ at infinity. To be precise, they studied zeroes inside the Siegel sets $\mathcal {D}_Y:=\{z\in \mathcal {D}{:}\,\text {Im}(z)\ge Y\}$ with $Y\longrightarrow \infty$ sufficiently fast along with $k\longrightarrow \infty$. Such a set can be regarded as a shrinking ball around the cusp at infinity as the hyperbolic area of $\mathcal {D}_Y$ equals 1/*Y* and so tends to zero as the weight tends to infinity. When $Y\gg k$ it is easy to see that there are no zeroes in $\mathcal {D}_Y$ except for the isolated simple zero at the cusp. Ghosh and Sarnak observed that, although the number of zeroes inside $\mathcal {D}_Y$ is proportional to the area of the domain, the statistical behaviour of the zeroes was very different from the uniform distribution when $\sqrt{k\log k}\ll Y\ll k$. Indeed, they observed that equidistribution of zeroes should not happen inside these sets and, based on numerical evidence and a random model, were led to conjecture that almost all of the zeroes inside $\mathcal {D}_Y$, for *Y* in the same range as before, concentrate on the half-lines $\text {Re}(s)=-1/2$ and $\text {Re}(s)=0$. They termed such zeroes to be “real” as the cusp form is itself real-valued on these lines.^1^ Ghosh and Sarnak conjectured that $50\%$ of the zeroes in these shrinking Siegel sets should lie on the line $\text {Re}(s)=-1/2$ and likewise $50\%$ on the line $\text {Re}(s)=0$. Furthermore, they obtained some results in this direction. In the end they were able to produce $\gg _\varepsilon (k/Y)^{1/2-1/40-\varepsilon }$ such real zeroes in the range $\sqrt{k\log k}\ll Y<k/100$. The exponent was later improved to $1/2-\varepsilon$ by Matomäki [37]. Producing real zeroes on the individual lines $\text {Re}(s)=-1/2$ and $\text {Re}(s)=0$ is more challenging, but Ghosh and Sarnak succeeded, the current best results, which are of polynomial growth, are again due to Matomäki [37]. Related to this Lester et al. [32] have also obtained some further results that we shall discuss below in more detail.

One may of course speculate that similar phenomenon holds also for other types of automorphic forms. The analogue of the holomorphic QUE conjecture is known for half-integral weight Hecke cusp forms thanks to the work of Lester and Radziwiłł [33] under the Generalised Riemann Hypothesis (GRH). Similarly to Rudnick’s work this implies the equidistribution of zeroes inside the fundamental domain $\Gamma _0(4)\backslash \mathbb H$, where $\Gamma _0(4)$ is the Hecke congruence subgroup of level 4, of course conditionally on GRH. Given this, it is natural to wonder whether the aforementioned results concerning the small scale distribution of zeroes generalise to these forms. The goal of the present paper is to address this as it seems that these questions have not been explored previously for half-integral weight Hecke cusp forms.

This is not that surprising as the half-integral weight situation has features that are not present in the setting of classical holomorphic cusp forms. Indeed, Ghosh and Sarnak (and subsequent works) exhibited a relationship between real zeroes of integral weight Hecke cusp forms and sign changes of their Fourier coefficients. We shall do the same in setting of half-integral weight Hecke cusp forms, but here there are certain key differences that make adapting the methods used in the integral weight setting challenging. In the classical case the methods of [12, 32, 37] used to study the behaviour of Fourier coefficients rely in a fundamental way on their multiplicativity. However, the Fourier coefficients of half-integral weight Hecke cusp forms lack this property, except at squares. Because of this the methods used in the previous works are not directly applicable in our setting and we have to use different tools to investigate the distribution of real zeroes.

**Fig. 1:**
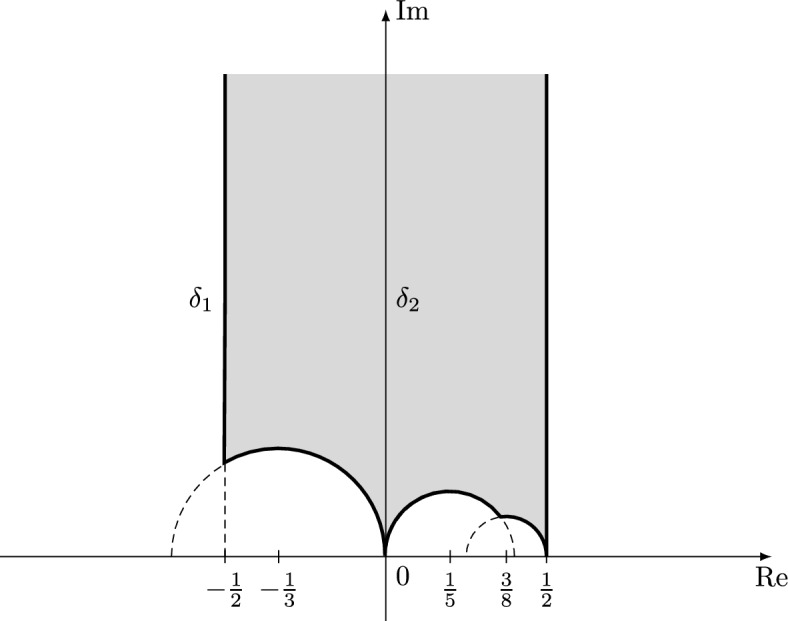
Fundamental domain for the action of the group $\Gamma _0(4)$ on $\mathbb H$

The fundamental domain $\mathcal {F}:=\Gamma _0(4)\backslash \mathbb H$ is taken to be the domain in the upper half-plane bordered by the vertical lines $\sigma =\pm \frac{1}{2}$ and circles^2^$B(-\frac{1}{3},\frac{1}{3})$, $B(\frac{1}{5},\frac{1}{5})$, and $B(\frac{3}{8},\frac{1}{8})$. Notice that this fundamental domain has three cusps at $\infty$, 0, and $\frac{1}{2}$ (these have widths 1, 4, and 1, respectively) (Fig. 1).

It is well-known that any half-integral weight Hecke cusp form may be normalised to have real Fourier coefficients^3^$c_g(n)$ and we impose this normalisation throughout the paper.

Analogous to the integral weight setting, we study the real zeroes, that is zeroes on the two geodesic segments

$$ \delta _1:=\left\{ s\in \mathbb C{:}\,\text {Re}(s)=-\frac{1}{2}\right\} \qquad \text {and} \qquad \delta _2:=\left\{ s\in \mathbb C{:}\,\text {Re}(s)=0\right\} $$

on which the cusp form takes real values in the normalisation above. Our first main result establishes that there are almost the expected amount (our result is optimal up to a power of the logarithm) of real zeroes for many half-integral weight Hecke cusp forms. Throughout the article, let *k* be a positive integer. We write $S_{k+\frac{1}{2}}(4)$ for the space of half-integral weight cusp forms of weight $k+\frac{1}{2}$ and level 4. Also, we denote by $S_{k+\frac{1}{2}}^+(4)\subset S_{k+\frac{1}{2}}(4)$ the Kohnen plus subspace and let $B^+_{k+\frac{1}{2}}$ denote a fixed Hecke eigenbasis for $S_{k+\frac{1}{2}}^+(4)$. Note that for half-integral weight cusp forms we cannot normalise the coefficient $c_g(1)$ to be equal to one without losing the algebraicity of the Fourier coefficients. This means that, unlike in the integral weight case, there is no canonical choice for $B_{k+\frac{1}{2}}^+$, but this causes no problems for us. We write $\mathcal {Z}(g):=\{z\in \mathcal {F}{:}\,g(z)=0\}$ for the set of zeroes of $g\in S_{k+\frac{1}{2}}^+(4)$ and let $\mathcal {F}_Y:=\{z\in \mathcal {F}{:}\,\text {Im}(z)\ge Y\}$. Finally, set^4^

$$ \mathcal {S}_K:=\bigcup _{k\sim K}B_{k+\frac{1}{2}}^+ $$

and note that the cardinality of this set is

$$ \sum _{k\sim K}\# B_{k+\frac{1}{2}}^+=\sum _{k\sim K}\#\mathcal {B}_k\sim \frac{K^2}{4} $$

as $\#\mathcal {B}_k\sim k/6$, where $\mathcal {B}_k$ is the Hecke eigenbasis for the space of holomorphic cusp forms of weight 2*k* and level one.

With these notations our first main result may be stated as follows.

### Theorem 1.1

Let *K* be a large parameter, $\varepsilon >0$ be an arbitrarily small fixed number, and $j\in \{1,2\}$. Then for $\gg _\varepsilon K^2/(\log K)^{3/2+\varepsilon }$ of the forms $g\in \mathcal {S}_K$ we have

$$ {\#}\{\mathcal {Z}(g)\cap \delta _j\cap \mathcal {F}_Y\}\gg \frac{K}{Y}\left( \log K\right) ^{-23/2} $$

for $\sqrt{K\log K}\le Y\le K^{1-\delta }$ with any small fixed constant $\delta >0$.

### Remark 1.2

Here we have not tried to optimise the powers of logarithm. The exponents present are conveniently chosen so to that the various exponents that appear in the proof are simple fractions.

As indicated above, the methods used in the integral weight case are hard to implement to our setting. However, building on the multiplicativity of the Fourier coefficients at squares, we show that for positive proportion of forms it is also possible to get some real zeroes.

### Theorem 1.3

Let *K* be a large parameter, $\varepsilon >0$ be an arbitrarily small fixed number, and $j\in \{1,2\}$. Then for at least $(1/2-\varepsilon )\#\mathcal {S}_K$ of the forms $g\in \mathcal {S}_K$ we have

$$ {\#}\{\mathcal {Z}(g)\cap \delta _j\cap \mathcal {F}_Y\}\gg \sqrt{\frac{K}{Y}} $$

for $\sqrt{K\log K}\le Y\le K^{1-\delta }$ with any small fixed constant $\delta >0$.

As far as the author is aware of, these are the first results concerning the small scale distribution of zeroes of Hecke cusp forms in the half-integral weight setting.^5^ Recall that the domain $\mathcal {F}_Y$ contains $\asymp k/Y$ zeroes of $g\in B_{k+\frac{1}{2}}^+$, at least conditionally on GRH. The above results give progress towards the conjecture^6^ that the number of zeroes of $g\in B^+_{k+\frac{1}{2}}$ is $\gg k/Y$ on $\delta _j\cap \mathcal {F}_Y$ for both of the individual lines $\delta _j$ in the same range of *Y* as before.^7^ That is, apart from $\log$-powers our first result shows that we get the expected number of real zeroes for many, albeit not a positive proportion of, forms. The above theorems may be compared to results of Lester et al. [32] who showed that in the setting of classical holomorphic Hecke cusp forms one has (for both $j\in \{1,2\})$

$$ \#\{\mathcal {Z}(f)\cap \delta _j\cap \mathcal {D}_Y\}\gg _\varepsilon \left( \frac{k}{Y}\right) ^{1-\varepsilon } $$

for all $\varepsilon >0$ under the Generalised Lindelöf Hypothesis, and unconditionally that for almost all forms one has

$$ \#\left\{ \mathcal {Z}(f)\cap \delta _j\cap \mathcal {D}_Y\right\} \asymp \frac{k}{Y} $$

in the same range of *Y* as above. However, as indicated above, our techniques used to prove Theorem 1.1 are very different compared to those used in the integral weight setting.

## The strategy

In this section we describe the main ideas that go into the proofs of the main theorems. Let *k* be a positive integer and let *g* be a Hecke cusp form of half-integral weight $k+\frac{1}{2}$ and level 4 that belongs to the Kohnen plus subspace. Every such *g* has a Fourier expansion

$$\begin{aligned} g(z)=\sum _{\begin{array}{c} n=1\\ (-1)^k n\equiv 0,1\,(\text {mod }4) \end{array}}^\infty c_g(n)n^{\frac{k}{2}-\frac{1}{4}}e(nz)\end{aligned}$$

with normalised real Fourier coefficients $c_g(n)$, which encode arithmetic information. For instance, Waldspurger’s formula shows that for fundamental discriminants *d* with $(-1)^kd>0$, $|c_g(|d|)|^2$ is proportional to the central value of an *L*-function, and so the magnitude of the *L*-function essentially determines the size of the coefficient $c_g(|d|)$. We shall use this fact repeatedly.

Let us then explain how to exploit information about the Fourier coefficients $c_g(n)$ in order to produce real zeroes. By a steepest descent argument it turns out that on the half-lines $\sigma +iy$, with $\sigma \in \{-\frac{1}{2},0\}$ and $y>0$, the values $g(\sigma +iy_\ell )$ for $y_\ell :=(k-1/2)/4\pi \ell$ and $\ell \in [c_1,c_2k/Y]$, for some absolute constants $c_1$ and $c_2$, are essentially determined by the single Fourier coefficient $c_g(\ell )$. Morally this reduces finding zeroes on these lines (i.e., real zeroes) to studying sign changes^8^ of Fourier coefficients $c_g(\ell )$. More precisely, we consider a dyadic subinterval $[X,2X]\subset [c_1,c_2k/Y]$ with $X\asymp k/Y$ and wish to show that $c_g(\ell )$ has many sign changes for many forms *g* as $\ell$ traverses over $\mathbb N\cap [X,2X]$.

The question of finding sign changes has been considered by many authors following the works of Knopp et al. [23] and Bruinier and Kohnen [8], the former of which showed that such forms have infinitely many sign changes. Subsequent works [17, 24, 30] showed that the sequence $\{c_g(n)\}_n$ exhibits many sign changes under suitable conditions. It is essential for our approach to obtain quantitative results that are uniform in the weight aspect.

Due to the lack of multiplicativity of these coefficients, the methods used to produce sign changes [12, 32, 37] are not readily available in our setting. For some special subsequences of coefficients, methods of multiplicative number theory are useful. For example, for a fixed positive squarefree integer *t* the coefficients $c_g(tm^2)$ are (essentially) multiplicative. This can be used in the proof of Theorem 1.3 as we now explain.

For this we rely on the following identity relating Fourier coefficients of half-integral weight Hecke cusp form *g* at certain arguments and the Fourier coefficients of its Shimura lift *f* at primes:

2.1 $$\begin{aligned} c_g(|d|p^2)=c_g(|d|)\left( \lambda _f(p)-\frac{\chi _d(p)}{\sqrt{p}}\right) . \end{aligned}$$

This is a special case of [25, Equation (2)]. Here *p* is any prime, *d* is a fundamental discriminant with $(-1)^kd>0$, $\lambda _f(p)$ are the Hecke eigenvalues of *f*, and $\chi _d$ is the unique real quadratic character of conductor |*d*|.

To benefit from this we have the following auxiliary result concerning the size of $c_g(|d|)$. For $g\in S_{k+\frac{1}{2}}^+(4)$ let us define

2.2 $$\begin{aligned} \alpha _g:=\frac{\Gamma \left( k-\frac{1}{2}\right) }{2(4\pi )^{k-\frac{1}{2}}\Vert g\Vert _2^2}, \end{aligned}$$

where the inner product $\Vert g\Vert _2^2:=\langle g,g\rangle$ is defined in (4.2). With this normalising factor the analogue of the Ramanujan–Petersson conjecture for the Fourier coefficients predicts that for a Hecke eigenform *g* we have $\sqrt{\alpha _g}c_g(m)\ll _\varepsilon k^{-1/2}(km)^\varepsilon$ with the implied constant depending only on $\varepsilon >0$.

### Proposition 2.1

Let *K* be a large parameter and $\varepsilon ,\,\theta >0$ be arbitrarily small, but fixed. Then for any fixed odd fundamental discriminant *d* with $|d|\ll 1$ we have $\sqrt{\alpha _g}|c_g(|d|)|> k^{-1/2-\theta }$ for at least $(1/4-\varepsilon )\#\mathcal {S}_K$ of the forms $g\in \mathcal {S}_K$ as $K\longrightarrow \infty$.

This follows by combining asymptotics for the mollified second and fourth moments of the Fourier coefficients $c_g(|d|)$ using the Cauchy–Schwarz inequality and removing the harmonic weights using an approach of Iwaniec and Sarnak [20] detailed in [2, 27]. The relevant moments are connected to moments of *L*-functions via Waldspurger’s formula and so the mollifier is constructed to counteract the large values of $L(1/2,f\otimes \chi _d)$. To be more specific, a Dirichlet series mollifier is a short linear form

$$ \sum _{\ell \le L}\frac{\lambda _f(\ell )\chi _d(\ell )x_\ell }{\sqrt{\ell }}, $$

where $L>0$ is the length of the mollifier and real numbers $x_\ell \ll \log L$ are chosen so that the mollifier mimics the behaviour of $L(1/2,f\otimes \chi _d)^{-1}$. Essentially the optimal choice for the coefficients $x_\ell$ translates to mollifier behaving approximately as

$$ \sum _{\ell \le L}\frac{\mu (\ell )\lambda _f(\ell )\chi _d(\ell )}{\sqrt{\ell }}\left( 1-\frac{\log \ell }{\log L}\right) $$

The moment results required are stated in two lemmas below. These have been obtained^9^ by Iwaniec and Sarnak [20, Theorem 3]. Throughout the text we set

$$ \omega _f:=\frac{2\pi ^2}{2k-1}\cdot \frac{1}{L(1,\text {sym}^2f)} $$

for $f\in S_{2k}(1)$ and write $\mathcal {B}_k$ for the Hecke eigenbasis of $S_{2k}(1)$, the space of holomorphic cusp forms of weight 2*k* and full level. It is useful to note that

2.3 $$\begin{aligned} \sum _{f\in \mathcal {B}_k}\omega _f\sim 1. \end{aligned}$$

Write $\mathcal {M}_{f,d}$ for the Iwaniec–Sarnak mollifier^10^ described in [20, p. 163].

### Lemma 2.2

Let *h* be a smooth compactly supported function on $\mathbb R_+$ taking non-negative values. Then, for the Iwaniec–Sarnak mollifier of length $L\le |d|^{-1}K(\log K)^{-20}$, there exists a small absolute constant $c>0$ so that uniformly in odd fundamental discriminants *d* with $|d|\le K^c$ we have

$$ \sum _{k\in \mathbb {Z}}h\left( \frac{2k}{K}\right) \sum _{f\in \mathcal {B}_k}\omega _f L\left( \frac{1}{2},f\otimes \chi _d\right) \mathcal {M}_{f,d}\sim \frac{K}{2}\int \limits _0^\infty h(t)\,\mathrm d t. $$

### Lemma 2.3

Let *h* be a smooth compactly supported function on $\mathbb R_+$ taking non-negative values. Then, for the Iwaniec–Sarnak mollifier of length $L\le |d|^{-1}K(\log K)^{-20}$, there exists a small absolute constant $c>0$ so that uniformly in odd fundamental discriminants *d* with $|d|\le K^c$ we have

$$ \sum _{k\in \mathbb {Z}}h\left( \frac{2k}{K}\right) \sum _{f\in \mathcal {B}_k}\omega _fL\left( \frac{1}{2},f\otimes \chi _d\right) ^2\mathcal {M}_{f,d}^2\sim K\left( \int \limits _0^\infty h(t)\,\mathrm d t\right) \left( 1+\frac{\log |d|K}{\log L} \right) . $$

Given these, in the beginning of Sect. 9 we explain that for a fixed odd fundamental discriminant *d* with $|d|\ll 1$, the number of forms $f\in \bigcup _{k\sim K} \mathcal {B}_{k}$ for which $\omega _f L(1/2,f\otimes \chi _d)> k^{-1-2\theta }$ is

$$ > \frac{1}{4}\left( 1-\frac{\log |d|}{\log K}\right) \sum _{k\sim K}\#\mathcal {B}_k. $$

But as the constraint $\omega _f L(1/2,f\otimes \chi _d)>k^{-1-2\theta }$ is equivalent to $\sqrt{\alpha _g}|c_g(|d|)>k^{-1/2-\theta }$ when *f* is the Shimura lift of *g* by Waldspurger’s formula, Proposition 2.1 follows.

From this we may conclude the proof of Theorem 1.3 as follows. In our argument the precise range of uniformity in *d* does not matter as we need these asymptotics for fixed $d\ll 1$. As alluded above, to find real zeroes it suffices to produce sign changes. Here the idea is to study sign changes of $c_g(|d|m^2)$ with $|d|\ll 1$ fixed and *m* traversing over odd natural numbers. We will show that for a positive proportion of the forms $g\in B_{k+\frac{1}{2}}^+$ one gets a positive proportion of sign changes for the sequence $c_g(|d|m^2)$ as *m* traverses over the odd integers in the dyadic interval [*X*, 2*X*] with $X\asymp \sqrt{K/|d|Y}$. This can be done by implementing arguments of Lester et al. [32] to (essentially) study sign changes of the multiplicative function $m\mapsto c_g(|d|)^{-1}c_g(|d|m^2)$ using (2.1). For this Proposition 2.1 provides important information about the size of $c_g(|d|)$ for a positive proportion of forms. This leads to the promised amount of sign changes for at least $(1/2-\varepsilon )\#\mathcal {S}_K$ of the forms in $\mathcal {S}_K$ by using different choices of *d* to treat forms $g\in B_{k+\frac{1}{2}}^+$ depending on the parity of *k* separately.

### Remark 2.4

As indicated above, for a fixed *d* we are able to study sign changes along the individual sequences $c_g(|d|m^2)$ as *m* varies. Ideally one would want to study the sign changes of the Fourier coefficients by varying both *m* and *d*. Note e.g. that for different fundamental discriminants *d* the sequences $\{|d|p^2\}_p$, with *p* traversing over the primes, are disjoint, but unfortunately it seems very hard to understand how these sequences are entangled.

Concerning the first main theorem it turns out that there is another way to produce sign changes along the fundamental discriminants that is not based on the multiplicativity of the Fourier coefficients. Recall that to find zeroes on individual lines $\delta _j$ it suffices (essentially) to detect sign changes of $c_g(m)$ along odd integers. For this we rely on the Shimura correspondence which attaches to $g\in S^+_{k+\frac{1}{2}}(4)$ a classical integral weight Hecke cusp form *f* of weight 2*k* and full level. Throughout the article we normalise the Shimura lift so that its first Fourier coefficient equals one.

For detecting sign changes we use an approach, which builds upon [34, 38]. We aim to obtain sign changes of $c_g(|d|)$ along squarefree $d\equiv 1\,(\text {mod }4)$, $(-1)^kd>0$, with $|d|\sim X$ where now $X\asymp K/Y$. The idea is to divide the dyadic interval [*X*, 2*X*] into short intervals $[x,x+H]$ with $1\le H\le x$ chosen as small as possible in terms of the weight so that we are able to detect sign changes and show that for many half-integral weight forms *g* it holds that for many $x\sim X$ such a short interval $[x,x+H]$ contains a sign change of $c_g(|d|)$.

To detect a sign change of $c_g(|d|)$ along such *d* with $(-1)^kd\in [x,x+H]$ it suffices to havewhere  means summing over squarefree integers $d\equiv 1$ (mod 4).

We also note that with the normalisation (2.2) Waldspurger’s formula [see (4.4) below] takes the form

2.4 $$\begin{aligned} \alpha _g|c_g(|d|)|^2=\omega _fL\left( \frac{1}{2},f\otimes \chi _d\right) , \end{aligned}$$

where the Shimura lift $f\in S_{2k}(1)$ is normalised so that $\lambda _f(1)=1$, for any fundamental discriminant *d* with $(-1)^k>0$. This is verified in Sect.4. Throughout the text the letter *g* is reserved for half-integral weight cusp forms and its Shimura lift is always denoted by the letter *f*. Because of (2.4) it is convenient to normalise by the factor $\sqrt{\alpha _g}$ and seek for the inequalitywhich naturally leads to a sign change of $c_g(|d|)$ on the interval $[x,x+H]$ as $\sqrt{\alpha _g}$ is a positive real number.

The following two estimates^11^ form a bulk of the proof of the first main theorem. Let us temporarily assume $X\asymp K/Y$ and let *H* be a parameter with $1\le H\le X$ to be specified later. Here we recall the assumption $\sqrt{K\log K}\ll Y\ll K^{1-\delta }$ from which it follows that $K^\delta \ll X\ll \sqrt{K/\log K}$.

### Proposition 2.5

We have

### Proposition 2.6

We havefor any $\varepsilon >0$.

Indeed, it is easy to see that the first statement implies that apart from $O(K^2/(\log K)^3)$ of the forms $g\in \mathcal {S}_K$ one has the property that the inequalityholds for almost all $x\sim X$ with the exceptional set having measure $\ll X/(\log X)^3$.

Likewise, the latter statement says that for $\gg _\varepsilon K^2/(\log K)^{3/2+\varepsilon }$ forms $g\in \mathcal {S}_K$ we have thatfor $x\gg X/(\log X)^{5/2}$ of $x\sim X$. Choosing, say, $H=(\log X)^9$ and combining the previous two observations, it follows that for $\gg _\varepsilon K^2/(\log K)^{3/2+\varepsilon }$ of the forms $g\in \mathcal {S}_K$ one has that for $\gg X/(\log X)^{5/2}$ of the numbers $x\sim X$ we have thatFrom these inequalities it is a simple matter to deduce a sign change of $c_g(|d|)$ with *d* an odd fundamental discriminant in the short intervals $[x,x+H]$. We refer to Sect. 8 for details. This leads to $\gg X/(\log X)^{5/2}H$ sign changes for the same number of forms as above, which consequently yields the promised number of real zeroes.

Both of the propositions above rely crucially on the sharp estimates for certain moments of quadratic twists of modular *L*-functions. The relevant results are the content of the following two lemmas.

### Lemma 2.7

Let $\phi$ and *h* be smooth compactly supported functions on $\mathbb R_+$. Thenfor any $\varepsilon >0$.

### Lemma 2.8

Let $\phi$ be a smooth compactly supported function on $\mathbb R_+$. Thenwhere the implicit constant depends on the weight functions *h* and $\phi$.

In the latter lemma and throughout the paper we write as an abuse of notation $\omega _g$ for $\omega _f$ when *f* is the Shimura lift of *g*.

Proofs of these results are reasonably standard. The first asymptotic formula is based on the half-integral weight variant of the Petersson formula for forms in the Kohnen plus subspace whereas the latter is a consequence of a large sieve inequality of Deshouillers and Iwaniec [10]. We remark that in the proof of Lemma 2.7 the summation over *k* plays no essential role and neither does the summation over the fundamental discriminants in the proof of Lemma 2.8. Actually with more work one could also evaluate the fourth moment in Lemma 2.8 asymptotically with main term $c\cdot XK\log (XK)$, where the constant *c* depends only on the weight function *h*. It is worth mentioning here that in such computation a nice structural feature that the off-diagonal contribution arising from an application of Petersson’s formula cancels part of the diagonal contribution from the application of the same formula is present. This gives a new instance of a similar phenomenon appearing in some prior works [4, 5, 22].

A few remarks are in order concerning the various averages in the preceding two lemmas and in particular to explain why they are required. Ideally we would like to evaluate^12^ the momentsfor $j\in \{2,4\}$ uniformly in terms of the weight $k+\frac{1}{2}$ of *g*. By Waldspurger’s formula these reduce to asymptotic evaluation of averaged first and second moments of $L(1/2,f\otimes \chi _{d})$ with uniformity in *k*. More precisely, we are required to understand the asymptotic behaviour of the sums2.5where $f\in S_{2k}(1)$ is the Shimura lift of *g*.

Recall that the complexity of a moment problem is measured by the ratio between logarithm of the analytic conductor and logarithm of the family size. Denote this ratio by *r*. The situations where $r=4$ is the edge of current technology where one can hope to obtain an asymptotic formula with a power saving error term. However, usually in this case we often barely fail to produce such asymptotics and a deep input is typically required in the rare cases when an asymptotic formula can be obtained. Note that in the latter sum in (2.5) the ratio between the logarithm of the conductor and the logarithm of the family size is greater than four. Moreover, in our applications the parameter *X* will be small, $X\ll \sqrt{K}$. In this situation the summation range is too short to evaluate even the first moment.

However, one can remedy the situation by introducing additional averages. Averaging over $g\in B_{k+\frac{1}{2}}^+$ brings us to the situation where the ratio *r* is precisely 4 in the second moment problem and in this case it is plausible that the methods from Li’s recent breakthrough [35] (which builds upon [50]) can be adapted to our setting to yield the expected asymptotics, but saving only a power of a logarithm in the error term. Moreover, a direct adaptation of this method cannot handle the introduction of a mollifier of length $\gg X^\varepsilon$. For all these reasons we have added one more averaging over the weights, which brings us to the situation where $2<r<3$.

### Possible extensions

It is natural to wonder whether we can improve the result of Theorem 1.1 to hold for a positive proportion of forms using a mollifier. By Waldspurger’s formula a natural choice for the mollifier to study sign changes of $c_g(|d|)$ would be a quantity $\mathcal {M}_g(d)$, which is a truncated Dirichlet series approximation for $L(1/2,f\otimes \chi _d)^{-1/2}$. A traditional approach would be to start by letting the mollifying factor for $c_g(|d|)$ to be of the form

$$\begin{aligned} \left( \sum _{\ell \le L}\frac{x_\ell \mu (\ell )\lambda _f(\ell )\chi _d(\ell )}{\sqrt{\ell }}\right) ^2 \end{aligned}$$

for some real coefficients $x_\ell$ to guarantee its positivity which is crucial for detecting sign changes. For this mollifier we would need to choose the coefficients $x_\ell$ so that

$$\begin{aligned} \sum _{\ell \le L}\frac{x_\ell \mu (\ell )\lambda _f(\ell )\chi _d(\ell )}{\sqrt{\ell }}\approx L\left( \frac{1}{2},f\otimes \chi _d\right) ^{-1/4}, \end{aligned}$$

but unfortunately it seems unclear how to do this effectively. Moreover, a crucial step in the proof of Proposition 2.5 would involve understanding sums of the form

2.6 $$\begin{aligned} \sum _{g\in B_{k+\frac{1}{2}}^+}\alpha _g c_g(|d|)c_g(|d|+h)\mathcal {M}_g(d)\mathcal {M}_g(d+h). \end{aligned}$$

In this case, after several applications of Hecke relations, studying (2.6) reduces to understanding the sums

$$ \sum _{g\in B_{k+\frac{1}{2}}^+}\alpha _g c_g(|d|)c_g(|d|+h)\lambda _f(\ell ). $$

This sum can be made amenable for Lemma 4.3 by inverting the relation (4.3) to write $c_g(|d|)\lambda _f(\ell )$ as a linear combination of Fourier coefficients of *g* at various arguments (at least for squarefree $\ell$). On the other hand, now evaluating the mollified momentsand optimising them becomes technically much more challenging compared to the corresponding moments with the Iwaniec–Sarnak mollifier $\mathcal {M}_{f,d}$ [20].

Related to this, again by a repeated application of Hecke relations, a key step towards evaluating these mollified moments is to evaluating the twisted analogues of the sums appearing in Lemmas 2.7 and 2.8. Indeed, with some additional work one may show that there exists an absolute constant $c>0$ so that uniformity in $\ell _1,\,\ell _2\le K^c$ one can obtain asymptotics forandHowever, it is hard to benefit from this due to lack of understanding on how choose the coefficients $x_\ell$ optimally in the mollifier.

A more promising approach, originating from [14, 44, 51], would be to use an Euler product mollifier as in the work of Lester and Radziwiłł [34]. This leads to major technical complications when computing the fourth moment of the Fourier coefficients. Nevertheless, it is likely that this approach can be pushed through and we will investigate this possibility in a future work. We emphasise that the purpose of the present article is to demonstrate that one is able to deduce highly non-trivial information about the zeroes of half-integral weight Hecke cusp forms using methods very different from those used in the integral weight case that do not produce strong results in the present setting.

### Organisation of the article

This paper is organised as follows. In Sect. 4 we gather basic facts about half-integral weight modular forms and other auxiliary results we need. In Sect. 5 we prove a result that reduces the question of finding real zeroes to studying sign changes of the Fourier coefficients. Lemmas 2.7 and 2.8 are proved in Sects. 6 and 7, respectively. These are then used in Sect. 8 to prove Propositions 2.5 and 2.6 from which Theorem 1.1 is deduced in the same section. In Sect. 9 Proposition 2.1 is proved and the proof of Theorem 1.3 is completed by studying sign changes of Fourier coefficients using ideas from multiplicative number theory.

## Notations

We use standard asymptotic notation. If *f* and *g* are complex-valued functions defined on some set, say $\mathcal {D}$, then we write $f\ll g$ to signify that $|f(x)|\leqslant C|g(x)|$ for all $x\in \mathcal {D}$ for some implicit constant $C\in \mathbb R_+$. The notation *O*(*g*) denotes a quantity that is $\ll g$, and $f\asymp g$ means that both $f\ll g$ and $g\ll f$. We write $f=o(g)$ if *g* never vanishes in $\mathcal {D}$ and $f(x)/g(x)\longrightarrow 0$ as $x\longrightarrow \infty$. Moreover, we write $f\sim g$ if $f(x)/g(x)\longrightarrow 1$ as $x\longrightarrow \infty$. The letter $\varepsilon$ denotes a positive real number, whose value can be fixed to be arbitrarily small, and whose value can be different in different instances in a proof. All implicit constants are allowed to depend on $\varepsilon$, on the implicit constants appearing in the assumptions of theorem statements, and on anything that has been fixed. When necessary, we will use subscripts $\ll _{\alpha ,\beta ,\ldots },O_{\alpha ,\beta ,\ldots }$, etc. to indicate when implicit constants are allowed to depend on quantities $\alpha ,\beta ,\ldots$

We define $\chi _d(\cdot ):=\left( \frac{d}{\cdot }\right)$, the Jacobi symbol, for all non-zero odd integers *d*. Let us also write $1_{m=n}$ for the characteristic function of the event $m=n$. Furthermore, $\text {Re}(s)$ and $\text {Im}(s)$ are the real- and imaginary parts of $s\in \mathbb C$, respectively, and occasionally we write $\sigma$ for $\text {Re}(s)$. We write $e(x):=e^{2\pi ix}$. For a compactly supported smooth function $\phi$, we define its Fourier transform $\widehat{\phi }(y)$ by

$$\begin{aligned} \widehat{\phi }(y):=\int \nolimits _\mathbb R \phi (x)e(-xy)\,\mathrm d x. \end{aligned}$$

The sum $\sum _{a\,(c)}^*$ means that the summation is over residue classes coprime to the modulus. Given coprime integers *a* and *c*, we write $\overline{a}\,(\text {mod } c)$ for the multiplicative inverse of *a* modulo *c*. As usual, $\Gamma$ denotes the Gamma function and $\mu$ denotes the Möbius function. Finally,  means we are summing over all odd fundamental discriminants.

## Preliminaries

### Half-integral weight forms

The group $\textrm{SL}_2(\mathbb R)$ acts on the upper half-plane $\mathbb H$ by $\gamma .z:=\frac{az+b}{cz+d}$, where $$\gamma =\begin{pmatrix} a & \quad b\\ c & \quad d \end{pmatrix}$$ and $z=x+iy\in \mathbb {C}$. Let $\Gamma _0(4)$ denote the congruence subgroup consisting of matrices $$\begin{pmatrix} a & \quad b\\ c & \quad d \end{pmatrix}$$ in $\textrm{SL}_2(\mathbb Z)$ such that *c* is divisible by 4.

Let $\theta (z):=\sum _{n=-\infty }^\infty e(n^2z)$ denote the standard theta function on $\mathbb H$. If $$A=\begin{pmatrix} a & \quad b\\ c & \quad d \end{pmatrix}\in \Gamma _0(4)$$, we have $\theta (Az)=j(A,z)\theta (z)$, where *j*(*A*, *z*) is the so-called theta multiplier. For an explicit formula for *j*(*A*, *z*), see [49, 1.10]. Fix a positive integer *k*. Let $S_{k+\frac{1}{2}}(4)$ denote the space of holomorphic cusp forms of weight $k+\frac{1}{2}$ for the group $\Gamma _0(4)$. This means that a function $g{:}\,\mathbb H\longrightarrow \mathbb C$ belongs to $S_{k+\frac{1}{2}}(4)$ if

- $$g(Az)=j(A,z)^{2k+1}g(z)$$ for every $$A\in \Gamma _0(4)$$.
- *g* is holomorphic.
- *g* vanishes at the cusps.

Any such form *g* has a Fourier expansion of the form

4.1 $$\begin{aligned} g(z)=\sum _{n=1}^\infty c_g(n)n^{\frac{k}{2}-\frac{1}{4}}e(nz), \end{aligned}$$

where $c_g(n)$ are the Fourier coefficients of *g*.

For $g,h\in S_{k+\frac{1}{2}}(4)$, we define the Petersson inner product $\langle g,h\rangle$ to be

4.2 $$\begin{aligned} \langle g,h\rangle :=\int \limits _{\Gamma _0(4)\backslash \mathbb H}g(z)\overline{h}(z)y^{k+\frac{1}{2}}\frac{\mathrm d x\,\mathrm d y}{y^2}. \end{aligned}$$

For any odd prime *p* there exists a Hecke operator $T(p^2)$ acting on the space of half-integral weight modular forms given by

$$ T(p^2)g(z):=\sum _{n=1}^\infty \left( c_g(p^2n)+\left( \frac{(-1)^k n}{p}\right) p^{k-1}c_g(n)+p^{2k-1}c_g\left( \frac{n}{p^2}\right) \right) e(nz). $$

Here we have used the convention that $c_g(x)=0$ unless $x\in \mathbb Z$. We call a half-integral weight cusp form a Hecke cusp form if $T(p^2)g=\gamma _g(p)g$ for all $p>2$ for some $\gamma _g(p)\in \mathbb C$.

The Kohnen plus subspace $S_{k+\frac{1}{2}}^+(4)\subset S_{k+\frac{1}{2}}(4)$ consists of all weight $k+\frac{1}{2}$ Hecke cusp forms whose $n^{\text {th}}$ Fourier coefficient vanishes whenever $(-1)^k n\equiv 2,3\,(\text {mod }4)$. This space has a basis consisting of simultaneous eigenfunctions of the $T(p^2)$ for odd *p*. As $k\longrightarrow \infty$, asymptotically one third of half-integral weight cusp forms lie in the Kohnen plus space by dimension considerations. In this space Shimura’s correspondence [49] between half-integral weight forms and integral weight forms is well-understood.

Indeed, Kohnen proved [26] that there exists a Hecke algebra isomorphism between $S^+_{k+\frac{1}{2}}(4)$ and the space of level 1 cusp forms of weight 2*k*. That is, $S^+_{k+\frac{1}{2}}(4)\simeq S_{2k}(1)$ as Hecke modules. Also recall that every Hecke cusp form $g\in S^+_{k+\frac{1}{2}}(4)$ can be normalised so that it has real Fourier coefficients and throughout the article we assume that *g* has been normalised in this way.

For a fundamental discriminant *d* with $(-1)^kd>0$ we know that

4.3 $$\begin{aligned} c_g(n^2|d|)=c_g(|d|)\sum _{r|n}\frac{\mu (r)\chi _d(r)}{\sqrt{r}}\lambda _f\left( \frac{n}{r}\right) , \end{aligned}$$

where $\lambda _f(n)$ denotes the $n{\text {th}}$ Hecke eigenvalue of the Shimura lift *f* (see equation (2) of [25]). In particular, if *n* is a prime *p* this reduces to (2.1).

The proof of our first main result uses the explicit form of Waldspurger’s formula due to Kohnen and Zagier [25].

#### Lemma 4.1

For a Hecke cusp form $g\in S^+_{k+\frac{1}{2}}(4)$ we have

4.4 $$\begin{aligned} |c_g(|d|)|^2=L\left( \frac{1}{2},f\otimes \chi _d\right) \cdot \frac{(k-1)!}{\pi ^k}\cdot \frac{\langle g,g\rangle }{\langle f,f\rangle } \end{aligned}$$

for each fundamental discriminant *d* with $(-1)^kd>0$, where *f* is a holomorphic modular form attached to *g* via the Shimura correspondence, normalised so that $\lambda _f(1)=1$.

Here for $f_1,f_2\in S_{2k}(1)$ the Petersson inner product, which is still denoted by $\langle f_1,f_2\rangle$, is defined to be

$$ \langle f_1,f_2\rangle :=\int \limits _{\textrm{SL}_2(\mathbb Z)\backslash \mathbb H}f_1(z)\overline{f_2}(z)y^{2k}\frac{\mathrm d x\,\mathrm d y}{y^2}. $$

We write $$\Vert f\Vert _2^2:=\langle f,f\rangle $$.

We also remark that $L(1/2,f\otimes \chi _d)$ vanishes when $(-1)^kd<0$ due to the sign in the functional equation. It follows directly from (4.4) that $L(1/2,f\otimes \chi _d)\ge 0$ otherwise. To verify the formula (2.4) it suffices the show that

4.5 $$\begin{aligned} \frac{\Gamma \left( k-\frac{1}{2}\right) }{2(4\pi )^{k-1/2}\langle g,g\rangle }\cdot \frac{(k-1)!}{\pi ^k}\cdot \frac{\langle g,g\rangle }{\langle f,f\rangle }=\frac{2\pi ^2}{2k-1}\cdot \frac{1}{L(1,\text {sym}^2f)}. \end{aligned}$$

To see this, we use a well-known identity (which follows from [42, (7)] and Legendre’s duplication formula)

$$ \langle f,f\rangle =\frac{\Gamma (k)\Gamma \left( k-\frac{1}{2}\right) (2k-1)}{2^{2k+1}\pi ^{2k+3/2}}\cdot L(1,\text {sym}^2f) $$

from which the assertion follows immediately.

### Tools from the theory of automorphic forms

Our first tool is an approximate functional equation for $L(1/2,f\otimes \chi _d)$. The following is an easy modification of [44, Lemma 5].

#### Lemma 4.2

Let *f* be a holomorphic Hecke cusp form of weight 2*k* for the full modular group $\textrm{SL}_2(\mathbb Z)$ and *d* be a fundamental discriminant with $(-1)^kd>0$. Then

$$\begin{aligned} L\left( \frac{1}{2},f\otimes \chi _d\right) =2\sum _{m=1}^\infty \frac{\lambda _f(m)\chi _d(m)}{\sqrt{m}}V_k\left( \frac{m}{|d|}\right) , \end{aligned}$$

where, for any $\sigma >2$,

4.6 $$\begin{aligned} V_k(x):=\frac{1}{2\pi i}\int \limits _{(\sigma )}g(s)x^{-s}e^{s^2}\frac{\mathrm d s}{s}\quad \text {with}\quad g(s):=(2\pi )^{-s}\frac{\Gamma (s+k)}{\Gamma (k)}. \end{aligned}$$

Furthermore, we have

$$\begin{aligned} V_k(\xi )=1+O\left( \frac{\xi }{k}\right) as\quad \xi \longrightarrow 0. \end{aligned}$$

We also have the estimates

$$\begin{aligned}&V_k(\xi )\ll _A \left( \frac{k}{\xi }\right) ^A, \\&V_k^{(B)}(\xi )\ll _{A,B}\xi ^{-B}\left( \frac{k}{\xi }\right) ^A \end{aligned}$$

for any $A>0$ and integer $B\ge 0$.

One of the most important tools is a half-integral weight analogue for the Petersson formula. The following result for forms in the Kohnen plus subspace is [6, Lemma 6] with a slightly different normalisation. Recall the definition of the normalising factor $\alpha _g$ from (2.2).

#### Lemma 4.3

Let $k\ge 3$ be an integer. Let *m*, *n* be positive integers with $(-1)^km,\,(-1)^kn\equiv 0,\,1\,(\text {mod }4)$. Then

$$\begin{aligned} \sum _{g\in B_{k+\frac{1}{2}}^+}\alpha _g c_g(m)c_g(n)=\frac{1}{3}\left( 1_{m=n}+2\pi e\left( -\frac{k+\frac{1}{2}}{4}\right) \sum _c\frac{K_{k+\frac{1}{2}}^+(m,n;c)}{c} J_{k-\frac{1}{2}}\left( \frac{4\pi \sqrt{mn}}{c}\right) \right) , \end{aligned}$$

where for $m,\,n\in \mathbb {Z},\,c\in \mathbb {N}$, and $\kappa \in \frac{1}{2}\mathbb {Z}$ we define the modified Kloosterman sum asHere

$$ \epsilon _d:={\left\{ \begin{array}{ll} 1 & \quad \text {if }d\equiv 1\,(4) \\ i & \quad \text {if }d\equiv 3\,(4). \end{array}\right. } $$

Note that the sum $K_\kappa ^+$ is 2-periodic in $\kappa$ and it satisfies a Weil-type bound (for this see [52], and for the general theory see [18, Chapter 11])

4.7 $$\begin{aligned} |K_\kappa ^+(m,n;c)|\le d(c)(m,n,c)^{1/2}c^{1/2}, \end{aligned}$$

where *d*(*c*) is the ordinary divisor function.

The large sieve is a key tool in the proof of Lemma 2.8. We employ the following variant due to Deshouillers and Iwaniec.

#### Lemma 4.4

Let $K\ge 1$, $N\ge 1/2$, and let $\{a_n\}$ be any sequence of complex numbers. Then we have

$$ \sum _{k\sim K}\sum _{f\in \mathcal {B}_k}\omega _f\left| \sum _{n\sim N}a_n\lambda _f(n)\right| ^2\ll _\varepsilon \left( K+K^{-1}N^{1+\varepsilon }\right) \sum _{n\sim N}|a_n|^2 $$

for any $$\varepsilon >0$$.

This follows from [10, Theorem 2] when taking the different normalisation into account. To check that the bound above is consistent with the one in [10], note that using the well-known identity

$$ \Gamma \left( k-\frac{1}{2}\right) =\frac{(2k-2)!}{4^{k-1}(k-1)!}\cdot \sqrt{\pi }$$

and (4.5) we have

$$\begin{aligned} \omega _f&=\frac{\Gamma \left( k-\frac{1}{2}\right) }{2(4\pi )^{k-1/2}\Vert f\Vert _2^2}\cdot \frac{(k-1)!}{\pi ^k}\\&=\frac{(2k-2)!}{4^{k-1}(k-1)!}\cdot \sqrt{\pi }\cdot \frac{(k-1)!}{2(4\pi )^{k-1/2}\pi ^k\Vert f\Vert _2^2}\\&=\frac{(2k-2)!}{(4\pi )^{2k-1}\Vert f\Vert _2^2}, \end{aligned}$$

which shows that $(2k-1)\omega _f$ corresponds to the normalising factor in [10, (1.28)] (when also taking into account the normalisation by $\Vert f\Vert _2^2$ which arises as in [10] the Hecke eigenbasis is orthonormal whereas in our case it is just orthogonal).

We make use of another large sieve inequality for the Fourier coefficients $\lambda _f(n)$. The following is a special case of [29, Theorem 1].

#### Lemma 4.5

Let $v\ge 1$ be a fixed integer and $2\le P<Q\le 2P$. Then

$$ \sum _{f\in \mathcal {B}_k}\left| \sum _{P<p\le Q}\frac{\lambda _f(p^v)}{p}\right| ^2\ll _v k\frac{1}{P\log P}+k^{10/11}\frac{Q^{v/5}}{(\log P)^2}. $$

We also recall the following result of Murty and Sinha [40, Theorem 2].

#### Lemma 4.6

Let *p* be a prime. Then for any interval $[\alpha ,\beta ]\subset [-2,2]$ we have

$$ \frac{\#\{f\in \mathcal {B}_k{:}\,\lambda _f(p)\in [\alpha ,\beta ]\}}{\#\mathcal {B}_k}=\int \limits _\alpha ^\beta \mathrm d \mu _p+O\left( \frac{\log p}{\log k}\right) , $$

where

$$ \mathrm d \mu _p:=\frac{p+1}{\pi }\cdot \frac{(1-x^2/4)^{1/2}}{\left( p^{1/2}+p^{-1/2}\right) ^2-x^2}\,\mathrm d x. $$

### Other tools

We also record the following well-known uniform estimate for the *J*-Bessel function [19, (2.11”)]. For $\nu \ge 0$ and $x>0$, the $J_\nu$-Bessel function satisfies the bound

4.8 $$\begin{aligned} J_\nu (x)\ll \frac{x}{\sqrt{\nu +1}}\left( \frac{ex}{2\nu +1}\right) ^\nu . \end{aligned}$$

Another key tool is the Poisson summation formula.

#### Lemma 4.7

Let *f* be a Schwartz function and *a* be a residue class modulo *c*. Then

$$ \sum _{n\equiv \,a\,(c)}f(n)=\frac{1}{c}\sum _n\widehat{f}\left( \frac{n}{c}\right) e\left( \frac{an}{c}\right) , $$

where $\widehat{f}$ denotes the Fourier transform of *f*. Note that this reduces to the classical Poisson summation formula when $c=1$.

We need two results from the theory of multiplicative functions. The first one is a special case of the Matomäki–Radziwiłł theorem [39, Theorem 1].

#### Lemma 4.8

([32, Lemma 3.2]) Let $f{:}\,\mathbb N\longrightarrow [-1,1]$ be a multiplicative function. Then there exists an absolute constant $C>1$ such that, for any $2\le y\le X$,

$$ \left| \frac{1}{y}\sum _{x\le n\le x+y}f(n)-\frac{1}{X}\sum _{n\sim X}f(n)\right| \le 2(\log y)^{-1/200} $$

for almost all $x\sim X$ with at most $CX(\log y)^{-1/100}$ exceptions.

Our final tool is a variant of Halász’s theorem (see [13, (6)]).

#### Lemma 4.9

Let $f{:}\,\mathbb N\longrightarrow [-1,1]$ be a multiplicative function. Then we have

$$ \frac{1}{X}\sum _{n\le X}f(n)\ll \exp \left( -\frac{1}{4}\sum _{p\le X}\frac{1-f(p)}{p}\right) . $$

## Reduction to the study of Fourier coefficients

Our approach to relate detecting real zeroes to properties of the Fourier coefficients follows the previous works [12, 32, 37] in the setting of integral weight cusp forms. The main result in this direction is the following observation, which is a half-integral weight analogue for [12, Theorem 3.1.].

### Proposition 5.1

Let $\alpha \in \{-\frac{1}{2},0\}$. Then there are positive constants $c_1,c_2$ and $\eta$ such that, for all integers $\ell \in ]c_1,c_2\sqrt{k/\log k}[$ and all Hecke eigenforms $g\in S^+_{k+\frac{1}{2}}(4)$, we have

$$\begin{aligned} \sqrt{\alpha _g}\left( \frac{e}{\ell }\right) ^{\frac{k}{2}-\frac{1}{4}}g(\alpha +iy_\ell )=\sqrt{\alpha _g}c_g(\ell )e(\alpha \ell )+O(k^{-1/2-\eta }), \end{aligned}$$

where $y_\ell :=(k-1/2)/4\pi \ell$ and the implicit constant in the error term is absolute.

The proof is the same as in [12]. The only automorphic input used in the proof, besides the existence of the Fourier expansion, is Deligne’s bound for the Hecke eigenvalues, an analogue of which is not known for half-integral weight forms. However, it turns out that the pointwise bound^13^$c(m)\ll _\varepsilon m^{1/6+\varepsilon }$ of Conrey and Iwaniec [9, Corollary 3] suffices once the *k*-dependence is made explicit. By the preceding footnote we have

5.1 $$\begin{aligned} \sqrt{\alpha _g}|c_g(m)|\ll _\varepsilon m^\varepsilon \sqrt{\alpha _g}|c_g(\tilde{m})|, \end{aligned}$$

where $\tilde{m}$ is the squarefree kernel of *m* and the implied constant depends only on $\varepsilon >0$. To estimate the latter factor on the right-hand side one combines the works of Young [53] and Petrow and Young [43] to see that for any squarefree^14^ integer *q* one has $L(1/2,f\otimes \chi _q)\ll _\varepsilon (kq)^{1/3+\varepsilon }$ uniformly in both *k* and *q*. Together with computations of Mao [3, Appendix 2] and a slight extension of these that appeared in [7, Section 9] this leads to the following pointwise bound.

### Lemma 5.2

Let $g\in S_{k+\frac{1}{2}}^+(4)$ be a Hecke eigenform. Then we have

5.2 $$\begin{aligned} \sqrt{\alpha _g}c_g(m)\ll _\varepsilon k^{-1/3+\varepsilon }m^{1/6+\varepsilon } \end{aligned}$$

for any $$\varepsilon >0$$.

Recall that here the most optimistic bound would be $\ll _\varepsilon k^{-1/2}(km)^\varepsilon$.

### Proof

In our normalisation [7, (9.1)] states that

$$ c_g(\tilde{m})\ll (k\tilde{m})^\varepsilon (4\pi )^{k/2+1/4}\langle g,g\rangle ^{1/2}\cdot \Gamma \left( k+\frac{1}{2}\right) ^{-1/2}L\left( \frac{1}{2},f\otimes \chi _{\tilde{m}}\right) ^{1/2}. $$

Now using (5.1), the relation $\Gamma (z+1)=z\Gamma (z)$, the estimate $L(1/2,f\otimes \chi _q)\ll _\varepsilon (kq)^{1/3+\varepsilon }$, and recalling the definition of $\alpha _g$ in (2.2), we have

$$\begin{aligned} \sqrt{\alpha _g}c_g(m)&\ll _\varepsilon m^\varepsilon \sqrt{\alpha _g}c_g(\tilde{m})\\&\ll _\varepsilon \frac{(km)^\varepsilon }{\langle g,g\rangle ^{1/2}}\langle g,g\rangle ^{1/2}\left( \frac{\Gamma \left( k-\frac{1}{2}\right) }{\Gamma \left( k+\frac{1}{2}\right) }\right) ^{1/2}L\left( \frac{1}{2},f\otimes \chi _{\tilde{m}}\right) ^{1/2}\\&\ll _\varepsilon (km)^\varepsilon k^{-1/2}(km)^{1/6}\\&\ll _\varepsilon k^{-1/3+\varepsilon }m^{1/6+\varepsilon }. \end{aligned}$$

Here we have also used the fact that as *g* is a Hecke eigenform it generates an irreducible cuspidal automorphic representation^15^ of $\widetilde{GL}_2(\mathbb A)$, the metaplectic cover of $\textrm{GL}_2(\mathbb A)$, and thus corresponds to one of the special forms (see the beginning of [3, Section 7.1]) for which [7, (9.1)] is valid. $\square$

The idea behind Proposition 5.1 is that the function $\xi \mapsto \xi ^{k}e^{-\xi }$ has a maximum at $\xi =k$ and is very localised there. We give a fairly detailed argument as the *k*-dependence in (5.2) is crucial and also remark that if the exponent of *k* in Lemma 5.2 would be large than $-1/12$, then the method used to obtain the required approximation (Proposition 5.1) would not succeed. This stems from treating the error term coming from Lemma 5.3 applied to the partial sum $\Phi _g^{(2)}$.

Let us set

$$\begin{aligned} I_s(y):= y^{(s-1)/2}e^{-y} \end{aligned}$$

for any $y>0$ and $s\in \mathbb C$. The following auxiliary result will be useful.

### Lemma 5.3

([12, Lemma 2.3]) For $|h|\ll k^{2/3-\delta }$, with any $\delta >0$, we have

$$I_{k+\frac{1}{2}}\left( \frac{k}{2}-\frac{1}{4}+h\right) =I_{k+\frac{1}{2}}\left( \frac{k}{2}-\frac{1}{4}\right) e^{-h^2/(k-1/2)}\left( 1+O\left( k^{-3\delta }\right) \right) .$$

With this at hand we are ready to prove the approximation of the values of *g* at certain arguments.

*Proof of Proposition* 5.1. Throughout the proof, let *y* be a real parameter with $\sqrt{k}\ll y<k/100$. Furthermore, let $\varepsilon >0$ be arbitrarily small but fixed. We freely refer to the argument of Ghosh and Sarnak for details. Just by the Fourier expansion (4.1) we have

$$\begin{aligned} \sqrt{\alpha _g}g(\alpha +iy)&=\sum _{m=1}^\infty \sqrt{\alpha _g}c_g(m)m^{\frac{k}{2}-\frac{1}{4}}e(m\alpha )e^{-2\pi my}\\&=(2\pi y)^{-\frac{k}{2}+\frac{1}{4}}\Phi _g(k;\alpha ,y), \end{aligned}$$

where

$$\begin{aligned} \Phi _g(k;\alpha ,y):&=\sum _{m=1}^\infty \sqrt{\alpha _g} c_g(m)e(m\alpha )(2\pi my)^{\frac{k}{2}-\frac{1}{4}}e^{-2\pi my}\\&=\sum _{m=1}^\infty \sqrt{\alpha _g}c_g(m)e(m\alpha )I_{k+\frac{1}{2}}(2\pi my). \end{aligned}$$

Let $1\le \Delta \ll k$ be a parameter specified later. We decompose $\Phi _g(k;\alpha ,y)$ into three pieces according to the size of *m*:

$$\begin{aligned} \Phi (k;\alpha ,y)&=\sum _{\begin{array}{c} m\ge 1\\ 2\pi my<\frac{k}{2}-\frac{1}{4}-\Delta \end{array}}\sqrt{\alpha _g}c_g(m)e(m\alpha )I_{k+\frac{1}{2}}(2\pi my)\\&\qquad \qquad +\sum _{|2\pi my-\frac{k}{2}+\frac{1}{4}|\le \Delta }\sqrt{\alpha _g}c_g(m)e(m\alpha )I_{k+\frac{1}{2}}(2\pi my)\\&\qquad \qquad +\sum _{2\pi my>\frac{k}{2}-\frac{1}{4}+\Delta }\sqrt{\alpha _g}c_g(m)e(m\alpha )I_{k+\frac{1}{2}}(2\pi my)\\&=:\Phi _g^{(1)}(k;\alpha ,y)+\Phi _g^{(2)}(k;\alpha ,y)+\Phi _g^{(3)}(k;\alpha ,y), \end{aligned}$$

say.

Starting with $\Phi _g^{(2)}(k;\alpha ,y)$, we shall apply Lemma 5.3. Choosing $h=2\pi my-(k/2-1/4)$ and $\delta =1/6-\varepsilon$ (anticipating the choice $\Delta \asymp \sqrt{k\log k})$ we have

$$ I_{k+\frac{1}{2}}(2\pi my)=I_{k+\frac{1}{2}}\left( \frac{k}{2}-\frac{1}{4}\right) e^{-|2\pi my-k/2+1/4|^2/(k-1/2)}\left( 1+O\left( k^{-3\delta }\right) \right) .$$

Using Lemma 5.2 the error term contributes to $\Phi _g^{(2)}(k;\alpha ,y)$ the amount

$$\begin{aligned}&\ll _\varepsilon k^{-1/3+\varepsilon }\left( \frac{k}{y}\right) ^{1/6+\varepsilon }I_{k+\frac{1}{2}}\left( \frac{k}{2}-\frac{1}{4}\right) \left( \sum _{|2\pi my-(k/2-1/4)|\le \Delta }1\right) k^{-3\delta }\\&\ll _\varepsilon k^{-2/3+\varepsilon }y^{-1/6}I_{k+\frac{1}{2}}\left( \frac{k}{2}-\frac{1}{4}\right) \left( 1+\frac{\Delta }{y}\right) \ll k^{-2/3+\varepsilon }y^{-1/6}I_{k+\frac{1}{2}}\left( \frac{k}{2}-\frac{1}{4}\right) \end{aligned}$$

as we shall choose $\Delta \asymp \sqrt{k\log k}$. Our aim now is to show that $\Phi ^{(1)}$ and $\Phi ^{(3)}$ both give a smaller contribution.

We first concentrate on $\Phi _g^{(3)}$. Using the identity $I_{s_1}(t)=t^{(s_1-s_2)/2}I_{s_2}(t)$ with $s_1=k+\frac{1}{2}$, $s_2=k+\frac{5}{6}+2\varepsilon$, and the estimate (5.2) we have

$$\begin{aligned} \Phi _g^{(3)}(g;\alpha ,y)&\ll _\varepsilon k^{-1/3+\varepsilon }\sum _{\begin{array}{c} m\ge 1\\ 2\pi my>\frac{k}{2}-\frac{1}{4}+\Delta \end{array}}m^{1/6+\varepsilon }(2\pi my)^{-1/6+\varepsilon }I_{k+\frac{5}{6}+2\varepsilon }(2\pi my)\\&\ll _\varepsilon y^{-1/6-\varepsilon }k^{-1/3+\varepsilon }\sum _{\begin{array}{c} m\ge 1\\ 2\pi my>\frac{k}{2}-\frac{1}{4}+\Delta \end{array}}I_{k+\frac{5}{6}+2\varepsilon }(2\pi my). \end{aligned}$$

Note that the function $t\mapsto I_{k+\frac{5}{6}+2\varepsilon }(t)$ achieves its maximum at $t=k/2-1/12+\varepsilon$. On the other hand, as $\Delta \ge 1$, the function $t\mapsto I_{k+\frac{5}{6}+2\varepsilon }(2\pi ty)$ is decreasing in the domain we are considering. Thus we may estimate the sum by an integral as

$$\begin{aligned} \Phi _g^{(3)}(k;\alpha ,y)&\ll _\varepsilon y^{-1/6-\varepsilon }k^{-1/3+\varepsilon }\\&\left( \int \limits _{(k/2-1/4+\Delta )/2\pi y}^\infty (2\pi yt)^{k/2-1/12+\varepsilon }e^{-2\pi yt}\,\mathrm d t+I_{k+\frac{5}{6}+2\varepsilon }\left( \frac{k}{2}-\frac{1}{4}+\Delta \right) \right) \\&\ll _\varepsilon y^{-1/6-\varepsilon }k^{-1/3+\varepsilon }\left( \frac{1}{y}\Gamma \left( \frac{k}{2}+\frac{11}{12}+\varepsilon ,\frac{k}{2}-\frac{1}{4}+\Delta \right) \right. \\&\left. +I_{k+\frac{5}{6}+2\varepsilon }\left( \frac{k}{2}-\frac{1}{4}+\Delta \right) \right) , \end{aligned}$$

where $\Gamma (s,x)$ is the incomplete Gamma function.

By using the Taylor expansion for $\log (1+x)$ we have

$$ I_{k+\frac{5}{6}+2\varepsilon }\left( \frac{k}{2}-\frac{1}{4}+\Delta \right) \ll _\varepsilon k^{1/6+\varepsilon }I_{k+\frac{1}{2}}\left( \frac{k}{2}-\frac{1}{4}\right) e^{-\Delta ^2/(k-1/2)}. $$

Indeed, we have

$$\begin{aligned} \frac{I_{k+\frac{5}{6}+2\varepsilon }\left( \frac{k}{2}-\frac{1}{4}+\Delta \right) }{I_{k+\frac{1}{2}}\left( \frac{k}{2}-\frac{1}{4}\right) }&=\left( \frac{k}{2}-\frac{1}{4}+\Delta \right) ^{1/6+\varepsilon }\left( 1+\frac{\Delta }{k/2-1/4}\right) ^{k/2-1/4}e^{-\Delta }\\&\ll _\varepsilon k^{1/6+\varepsilon }\exp \left( \Delta -\frac{\Delta ^2}{k-1/2}\right) e^{-\Delta }\\&\ll _\varepsilon k^{1/6+\varepsilon }e^{-\Delta ^2/(k-1/2)}, \end{aligned}$$

where we have used the Taylor expansion of $\log (1+x)$ in the form

$$\begin{aligned} 1+\frac{\Delta }{k/2-1/4}&=\exp \left( \log \left( 1+\frac{\Delta }{k/2-1/4}\right) \right) \\&=\exp \left( \frac{\Delta }{k/2-1/4}-\frac{\Delta ^2}{2(k/2-1/4)^2}+O\left( k^{-1/2}\right) \right) . \end{aligned}$$

Here the last estimate follows as we are going to choose $\Delta \asymp \sqrt{k\log k}$. This gives the desired estimate.

Estimating the incomplete Gamma function using a result of Natalini and Palumbo (see [41]) as in [12] with the choices $a=k/2+11/2+\varepsilon$, $x=k/2-1/4+\Delta$, and $\sigma =1+(k/2-1/4)/\Delta +\varepsilon '$, we have

$$\begin{aligned} \Gamma \left( \frac{k}{2}+\frac{11}{12}+\varepsilon ,\frac{k}{2}-\frac{1}{4}+\Delta \right)&\ll _\varepsilon \left( 1+\frac{k}{\Delta }+\varepsilon \right) \left( \frac{k}{2}-\frac{1}{4}+\Delta \right) ^{\frac{k}{2}-\frac{1}{12}+\varepsilon }e^{-\frac{k}{2}+\frac{1}{4}-\Delta }\\&\ll _\varepsilon \frac{k}{\Delta }e^{-\Delta ^2/(k-1/2)}k^{1/6+\varepsilon } I_{k+\frac{1}{2}}\left( \frac{k}{2}-\frac{1}{4}\right) . \end{aligned}$$

Combining all the preceding estimates we have shown that

$$\begin{aligned} \Phi _g^{(3)}(g;\alpha ,y)\ll _\varepsilon k^{-1/6+\varepsilon }y^{-1/6-\varepsilon }I_{k+\frac{1}{2}}\left( \frac{k}{2}-\frac{1}{4}\right) e^{-\Delta ^2/(k-1/2)}\left( 1+\frac{1}{y}\cdot \frac{k}{\Delta }\right) . \end{aligned}$$

Choosing $\Delta =\sqrt{A(k-1/2)\log k}$ for a sufficiently large fixed constant $A>0$ the right-hand side of the previous estimate is

$$ \ll _\varepsilon k^{-1/6-A+\varepsilon }y^{-1/6}I_{k+\frac{1}{2}}\left( \frac{k}{2}-\frac{1}{4}\right) . $$

For $\Phi _g^{(1)}$ note that

$$ \sqrt{\alpha _g}c_g(m)\ll _\varepsilon k^{-1/3+\varepsilon }m^{1/6+\varepsilon }\ll _\varepsilon k^{- 1/6+\varepsilon }y^{-1/6-\varepsilon }. $$

In addition, the function $t\mapsto I_{k+\frac{1}{2}}(2\pi ty)$ is strictly increasing in the interval we are considering, and so we may again approximate the sum by an integral as

5.3 $$\begin{aligned}&\Phi _g^{(1)}(k;\alpha ,y)\ll _\varepsilon k^{-1/6+\varepsilon }y^{-1/6-\varepsilon }\nonumber \\&\quad \times \left( \int \limits _1^{(k/2-1/4-\Delta )/2\pi y}(2\pi yt)^{k/2-1/4}e^{-2\pi yt}\,\mathrm d t+I_{k+\frac{1}{2}}(2\pi y)+I_{k+\frac{1}{2}}\left( \frac{k}{2}-\frac{1}{4}-\Delta \right) \right) . \end{aligned}$$

Now by the definition of $I_s(y)$ we have

$$ I_{k+\frac{1}{2}}(2\pi y)=I_{k+\frac{1}{2}}\left( \frac{k}{2}-\frac{1}{4}\right) \left( \frac{2\pi ye}{k/2-1/4}\right) ^{k/2-1/4}e^{-2\pi y}, $$

which decays exponentially when $y<k/100$.

As above, using the Taylor series approximation of $\log (1-x)$ we have

$$ I_{k+\frac{1}{2}}\left( \frac{k}{2}-\frac{1}{4}-\Delta \right) \ll I_{k+\frac{1}{2}}\left( \frac{k}{2}-\frac{1}{4}\right) e^{-\Delta ^2/(k-1/2).} $$

The integral in (5.3) is estimated by splitting it into two parts. Let $\Delta _1>\Delta$ be a parameter specified later, and write

$$\begin{aligned}&\int \limits _1^{(k/2-1/4+\Delta )/2\pi y}(2\pi yt)^{k/2-1/4}e^{-2\pi yt}\,\mathrm d t\\&\quad =\left( \int \limits _1^{(k/2-1/4-\Delta _1)/2\pi y}+\int \limits _{(k/2-1/4-\Delta _1)/2\pi y}^{(k/2-1/4-\Delta )/2\pi y}\right) (2\pi yt)^{k/2-1/4}e^{-2\pi yt}\,\mathrm d t. \end{aligned}$$

By making the change of variables $t\mapsto \left( \frac{k}{2}-\frac{1}{4}\right) t/2\pi y$ this simplifies into

$$\begin{aligned}&\frac{\left( \frac{k}{2}-\frac{1}{4}\right) ^{k/2+3/4}}{2\pi y}\left( \int \limits _{2\pi y/(k/2-1/4)}^{1-\Delta _1/(k/2-1/4)}+\int \limits _{1-\Delta _1/(k/2-1/4)}^{1-\Delta /(k/2-1/4)}\right) t^{k/2-1/4}e^{-\left( \frac{k}{2}-\frac{1}{4}\right) t}\,\mathrm d t\\&\quad =\frac{\left( \frac{k}{2}-\frac{1}{4}\right) }{2\pi y}I_{k+\frac{1}{2}}\left( \frac{k}{2}-\frac{1}{4}\right) \left( \int \limits _{2\pi y/(k/2-1/4)}^{1-\Delta _1/(k/2-1/4)}+\int \limits _{1-\Delta _1/(k/2-1/4)}^{1-\Delta /(k/2-1/4)}\right) t^{k/2-1/4}e^{\left( \frac{k}{2}-\frac{1}{4}\right) (1-t)}\,\mathrm d t. \end{aligned}$$

As the integrand is strictly increasing, the latter integral is

$$\begin{aligned} \ll \frac{\Delta _1-\Delta }{k}\left( 1-\frac{\Delta }{k/2-1/4}\right) ^{k/2-1/4}e^\Delta \ll \frac{\Delta _1-\Delta }{k}e^{-\Delta ^2/(k-1/2)}, \end{aligned}$$

where we have again used the Taylor expansion of $\log (1-x)$ in the latter estimate.

Similarly, estimating by absolute values and using the Taylor expansion the other part of the integral is $\ll e^{-\Delta _1^2/(k-1/2)}$ provided that $\Delta _1=o(k^{2/3})$, which will be satisfied as we shall choose $\Delta _1:=\sqrt{B(k-1/2)\log k}$ for a fixed constant $B>A+\frac{1}{2}$. With this choice, gathering all the estimates, we have

$$\begin{aligned} \Phi _g^{(1)}(k;\alpha ,y)&\ll _\varepsilon k^{-1/6+\varepsilon }y^{-1/6-\varepsilon }I_{k+\frac{1}{2}}\left( \frac{k}{2}-\frac{1}{4}\right) \frac{k}{y}\left( \frac{\Delta _1-\Delta }{k}e^{-\Delta ^2/(k-1/2)}+e^{-\Delta _1^2/(k-1/2)}\right) \\&\ll _\varepsilon k^{-1/6+\varepsilon }y^{-1/6-\varepsilon }I_{k+\frac{1}{2}}\left( \frac{k}{2}-\frac{1}{4}\right) \frac{k}{y}\left( \frac{\sqrt{k\log k}}{k} k^{-A}+k^{-B}\right) \\&\ll _\varepsilon k^{-1/6-A+\varepsilon }y^{-1/6-\varepsilon }I_{k+\frac{1}{2}}\left( \frac{k}{2}-\frac{1}{4}\right) , \end{aligned}$$

where the last step follows from recalling that $B>A+1/2$ and choosing *A* to be sufficiently large.

In total we have shown that

$$\begin{aligned}&\sqrt{\alpha _g}g(\alpha +iy)\\&\quad =(2\pi y)^{-\frac{k}{2}+\frac{1}{4}}I_{k+\frac{1}{2}} \left( \frac{k}{2}-\frac{1}{4}\right) \\&\qquad \,\,\bigg (\sum _{\begin{array}{c} m\ge 1\\ |2\pi my-(k/2-1/4)|\le \Delta \end{array}}\sqrt{\alpha _g}c_g(m)e(m\alpha )e^{-|2\pi my-(k/2-1/4)|^2/(k-1/2)}\\&\qquad \qquad +O_\varepsilon \left( k^{-2/3+\varepsilon }y^{-1/6}\right) \bigg ). \end{aligned}$$

Now observe that for $y_\ell =(k-1/2)/4\pi \ell$,

$$ \left| 2\pi my_\ell -\left( \frac{k}{2}-\frac{1}{4}\right) \right| \le \Delta \quad \Longleftrightarrow \quad |m-\ell |\le \frac{\Delta \ell }{k/2-1/4}, $$

which forces $m=\ell$ in the light of the choice for $\Delta$ when $\ell \le \sqrt{(k/2-1/4)/(2A\log k)}$.

Hence, by recalling that $y\gg \sqrt{k}$,

$$\begin{aligned} \sqrt{\alpha _g}g(\alpha +iy_\ell )&=(2\pi y_\ell )^{-\frac{k}{2}+\frac{1}{4}}I_{k+\frac{1}{2}}\left( \frac{k}{2}-\frac{1}{4}\right) \bigg (\sqrt{\alpha _g}c_g(\ell )e(\alpha \ell )+O\left( k^{-1/2-\eta }\right) \bigg )\\&=\left( \frac{\ell }{e}\right) ^{\frac{k}{2}-\frac{1}{4}}\bigg (\sqrt{\alpha _g}c_g(\ell )e(\alpha \ell )+O\left( k^{-1/2-\eta }\right) \bigg ) \end{aligned}$$

for, say, $\eta =1/4-\varepsilon$ for any sufficiently small fixed $\varepsilon >0$. This completes the proof. $\square$

## Proof of Lemma 2.7

In this section we evaluate the average of the absolute squares of Fourier coefficients of half-integral weight Hecke cusp forms.

Recall that the sum we aim to evaluate isExecuting the *g*-sum using Lemma 4.3 and estimating trivially this equalsThe *J*-Bessel function is exponentially small when $c>100|d|/k$ by (4.8). Recalling that $|d|\le X\ll \sqrt{K/\log K}$ and $k\asymp K$ this leads tousing the Weil bound (4.7).

To evaluate the main term, we begin by detecting the condition that *d* is squarefree by means of the identity

6.1 $$\begin{aligned} \sum _{\begin{array}{c} \alpha =1\\ \alpha ^2|d \end{array}}^\infty \mu (\alpha )={\left\{ \begin{array}{ll} 1 & \quad d\text { is squarefree}\\ 0 & \quad \text {otherwise} \end{array}\right. } \end{aligned}$$

This together with the Poisson summation (Lemma 4.7) and noting that the compact support of $\phi$ restricts the $\alpha$-sum to $\alpha \ll \sqrt{X}$ givesThe main contribution arises when $m=0$. Noting that

$$ \sum _{\begin{array}{c} \alpha =1\\ (\alpha ,2)=1 \end{array}}^\infty \frac{\mu (\alpha )}{\alpha ^2}=\frac{8}{\pi ^2},\qquad \qquad \sum _{\begin{array}{c} \alpha \gg \sqrt{X}\\ (\alpha ,2)=1 \end{array}}\frac{\mu (\alpha )}{\alpha ^2}\ll _\varepsilon X^{-1/2+\varepsilon } $$

for any $\varepsilon >0$, and summing the *k*-sum using Poisson summation we conclude that the $m=0$ contribution is given by

6.2 $$\begin{aligned} \frac{2XK}{3\pi ^2}\widehat{h}(0)\widehat{\phi }(0)+O_\varepsilon \left( KX^{1/2+\varepsilon }\right) . \end{aligned}$$

For the terms with $m\ne 0$, integrating by parts twice we see that the exponential integral is bounded by $\ll (\alpha ^2/mX)^2$ and thus these terms contribute an amount

$$\begin{aligned}&\ll X\sum _{\alpha \ll \sqrt{X}}\frac{1}{\alpha ^2}\sum _{m\ne 0}\frac{\alpha ^4}{(mX)^2}\\&\ll \frac{1}{X}\sum _{\alpha \ll \sqrt{X}}\alpha ^2\\&\ll \sqrt{X} \end{aligned}$$

to the *d*-sum. Now estimating the *k*-sum trivially completes the proof. $\square$

## Proof of Lemma 2.8

We now move to the proof of the second lemma. Recall that the sum we are considering isUsing the relation (2.4) our sum takes the formand applying the approximate functional equation (Lemma 4.2) bounds this from the above byNow applying Lemma 4.4, recalling that $|d|\ll X\ll \sqrt{K}$, and estimating the *d*-sum trivially yields an upper boundThis completes the proof. $\square$

## Proof of Theorem 1.1

We first prove Theorem 1.1 assuming the truth of Propositions 2.5 and 2.6. Let $\alpha \in \{-\frac{1}{2},0\}$, $\varepsilon >0$ be any fixed small constant, and let *K* be large positive parameter. Recall that as $g(\alpha +iy)$ is real-valued for these values of $\alpha$, Proposition 5.1 yields information on the zeroes inside the Siegel sets

$$ \mathcal {F}_Y=\{z\in \Gamma _0(4)\backslash \mathbb H{:}\, \text {Im}(z)\ge Y\} $$

with $c_1'\sqrt{k\log k}\le Y\le c_2'k$ for some positive constants $c_1'$ and $c_2'$. Let $\eta$ be as in Proposition 5.1. It follows immediately from that result that if we can find numbers $\ell _1,\ell _2\in ]c_1,c_2k/Y[$ so that

8.1 $$\begin{aligned} \sqrt{\alpha _g}c_g(\ell _1)e(\alpha \ell _1)<-k^{-\delta }<k^{-\delta }<\sqrt{\alpha _g}c_g(\ell _2)e(\alpha \ell _2) \end{aligned}$$

for some $\delta <1/2+\eta$, then *g*(*z*) has a zero $\alpha +iy$ with *y* between $y_{\ell _1}$ and $y_{\ell _2}$. Observe that

$$\begin{aligned} e(\alpha \ell )={\left\{ \begin{array}{ll} 1 & \quad \text {if }\alpha =0\\ (-1)^\ell & \quad \text {if }\alpha =-1/2 \end{array}\right. } \end{aligned}$$

Hence, in order to find real zeroes on the line $\text {Re}(s)=0$ it suffices (essentially) to detect sign changes among the Fourier coefficients whereas on the line $\text {Re}(s)=-1/2$ one needs to find pairs $(\ell _1,\ell _2)$ with $\ell _i$ odd for which (8.1) holds. As we restrict to odd fundamental discriminants *d* for which $(-1)^k d>0$, we automatically obtain real zeroes on both of the individual geodesic segments $\text {Re}(s)=-1/2$ and $\text {Re}(s)=0$.

Remember that in order to detect sign changes along the sequence $d\equiv 1\,(\text {mod }4)$ with *d* squarefree and $(-1)^kd>0$ in the short interval $[x,x+H]$, $x\sim X\asymp K/Y$, it suffices to haveChoose $H=(\log X)^9$. With this choice it follows easily from Propositions 2.5 and 2.6 that for $\gg _\varepsilon K^2/(\log K)^{3/2+\varepsilon }$ of the forms $g\in \mathcal {S}_K$ we have thatholds for $\gg X/(\log X)^{5/2}$ of the numbers $x\sim X$. We note that the contribution coming from the summands with $\sqrt{\alpha _g}|c_g(|d|)|\le k^{-\delta }$ is trivially bounded by

$$\ll \sum _{\begin{array}{c} x\le (-1)^k d\le x+H\\ \sqrt{\alpha _g} |c_g(|d|)|\le k^{-\delta } \end{array}}\sqrt{\alpha _g}|c_g(|d|)|\le 2HK^{-\delta }$$

and so we conclude, choosing $\delta =1/2+\eta /2$ for concreteness so that $\delta >1/2$, that for the same proportion of $g\in \mathcal {S}_K$ and $x\sim X$ we haveNow observe that this impliesSimilarly,Thus we have shown that for $\gg _\varepsilon K^2/(\log K)^{3/2+\varepsilon }$ of the forms $g\in \mathcal {S}_K$ the short interval $[x,x+H]$, $x\sim X$, contains numbers $(-1)^k d_\pm$, with both $d_\pm$ odd fundamental discriminants, for which $\sqrt{\alpha _g}c_g(|d_+|)>k^{-\delta }$ and $\sqrt{\alpha _g}c_g(|d_-|)<-k^{-\delta }$, for $\gg X/(\log X)^{5/2}$ of the numbers $x\sim X$. As discussed above, this leads to

$$ \gg \frac{X}{(\log X)^{5/2}H}\asymp \frac{K}{Y(\log X)^{23/2}} $$

real zeroes on both of the line segments $\delta _1$ and $\delta _2$, as claimed. $\square$

Next we shall show how the above two propositions just applied follow from Lemmas 2.7 and 2.8. We start by proving the first key proposition.

### Proof of Proposition 2.5

Recall that

$$ \mathcal {S}_K=\bigcup _{k\sim K}B_{k+\frac{1}{2}}^+. $$

Denoteand

$$ \mathcal {T}_{1,g}(X;H):=\#\left\{ x\sim X{:}\,S_{1,g}(x;H)\ge \sqrt{H} \cdot k^{-1/2}(\log K)^3\right\} . $$

Choose *h* to be a non-negative smooth function that is supported in the interval [1/2, 5/2] and is identically one in [1, 2]. With the above notation the quantity we need to bound is by Markov’s inequality8.2By opening the absolute square the inner sum over $x\sim X$ can be rearranged intoLet us first focus on the diagonal terms with $d_1=d_2$. In this case the total contribution to (8.2) is given byAdding a smooth weight function that localises $(-1)^kd\sim X$ and applying Lemma 2.7 this is bounded by

$$\begin{aligned}&\ll K^2\frac{(\log X)^3}{(\log K)^6}\ll \frac{K^2}{(\log K)^3}, \end{aligned}$$

as desired.

Let us then focus on the off-diagonal corresponding to the terms with $d_1\ne d_2$. We apply Lemma 4.3 to see that8.3But using the uniform estimate (4.8), the Weyl bound (4.7) and noting that $|d_1|$, $|d_2|\asymp X$, the inner sum may be bounded as

$$\begin{aligned}&\sum _{4|c}\frac{K_{k+\frac{1}{2}}^+(|d_1|,|d_2|;c)}{c} \cdot i^{k} J_{k-\frac{1}{2}}\left( \frac{4\pi \sqrt{|d_1d_2|}}{c}\right) \\&\ll _\varepsilon \sum _{4|c}\frac{c^{1/2+\varepsilon }X^{1/2}}{c}\cdot \frac{\sqrt{|d_1d_2|}}{c}\cdot \frac{1}{\sqrt{k}}\left( \frac{2\pi e\sqrt{|d_1d_2|}}{ck}\right) ^{k-1/2}\\&\quad \ll _\varepsilon \frac{X^{3/2}}{\sqrt{k}}\left( \frac{2\pi e\sqrt{|d_1d_2|}}{k}\right) ^{k-1/2}\sum _{4|c}\frac{1}{c^{1+k-\varepsilon }}\\&\quad \ll \frac{X^{3/2}}{\sqrt{k}}\left( \frac{2\pi e\sqrt{|d_1d_2|}}{k}\right) ^{k-1/2} . \end{aligned}$$

Now recall that $X\ll \sqrt{K}$ and $k\asymp K$ to see that the latter factor on the right-hand side decays exponentially. Thus estimating all the other sums in (8.3) trivially shows that the off-diagonal contribution is

$$\begin{aligned} \ll \frac{K}{HX}\cdot \frac{X^{3/2}}{\sqrt{K}}\cdot KXH^2\left( K^{-1/4}\right) ^{K-1/2}, \end{aligned}$$

which is negligible in the above range of *X*. This completes the proof. $\square$

### Proof of Proposition 2.6

We start by deriving a lower bound for the weighted sum of the terms $|c_g(|d|)|$ on average over the forms $g\in \mathcal {S}_K$. Applying Hölder’s inequality we havewhere *h* is a smooth compactly supported function that minorises the characteristic function of the interval [1, 2] and satisfies $\widehat{h}(0)\ne 0$.

Using Lemmas 2.7 and 2.8 for the second and fourth moments of the Fourier coefficients respectively we obtainOn the other hand, by Lemma 2.7 and Waldspurger’s formula we also haveIn the first estimate we have used (2.4) in the form

$$\begin{aligned} \alpha _g^{1/2}\omega _g^{1/2}|c_g(|d|)|&=\omega _gL\left( \frac{1}{2},f\otimes \chi _d\right) ^{1/2}\\&=\frac{\log XK}{\log XK}\omega _g L\left( \frac{1}{2},f\otimes \chi _d\right) ^{1/2}\\&<(\log XK)^{-1}\omega _g L\left( \frac{1}{2},f\otimes \chi _d\right) \\&=(\log XK)^{-1}\alpha _g|c_g(|d|)|^2 \end{aligned}$$

when $$L(1/2,f\otimes \chi _d)>(\log XK)^2$$.

From this we infer the lower boundLet us now define the setFrom the work above it follows thatwhere we have used the relation $\omega _g^{1/2}\alpha _g^{1/2}|c_g(|d|)|=\omega _g\sqrt{L(1/2,f\otimes \chi _d)}$ in the last estimate. Using an easy estimate $\sum _{g\in B_{k+\frac{1}{2}}^+}\omega _g^{1/2}\ll \sqrt{k}$ (which follows from the Cauchy–Schwarz inequality and (2.3)) we conclude that

8.4 $$\begin{aligned} \sum _{k\in \mathbb {Z}}h\left( \frac{k}{K}\right) \sum _{g\in B_{k+\frac{1}{2}}^+}\omega _g\left| \mathcal {V}_g\right| \gg \frac{KX}{(\log XK)^{3/2}}. \end{aligned}$$

To remove the harmonic weights $\omega _g$ note that by [2, (8.18)] we have

$$\begin{aligned} \sum _{g\in B_{k+\frac{1}{2}}^+}|\mathcal {V}_g|\gg K\sum _{g\in B_{k+\frac{1}{2}}^+}\omega _g|\mathcal {V}_g|L(1,\text {sym}^2f). \end{aligned}$$

As there exists $A>0$ so that $L(1,\text {sym}^2f)\gg (\log \log K)^{-A}$ for all but $O(K^\varepsilon )$ Hecke eigenforms $f\in \mathcal {B}_{k}$ (see [31]) and we have the Hoffstein–Lockhart bounds $1/\log k\ll L(1,\text {sym}^2f)\ll \log k$, it follows from (8.4) that

8.5 $$\begin{aligned} \sum _{k\in \mathbb {Z}}h\left( \frac{k}{K}\right) \sum _{g\in B_{k+\frac{1}{2}}^+}\left| \mathcal {V}_g\right| \gg _\varepsilon \frac{XK^2}{(\log XK)^{3/2+\varepsilon }}. \end{aligned}$$

Let us introduce the set

$$ \mathcal {U}:=\left\{ g\in \mathcal {S}_K{:}\,|\mathcal {V}_g|>\frac{X}{(\log X)^{5/2}}\right\} . $$

Now from (8.5) we deduce that

$$\begin{aligned} \frac{XK^2}{(\log XK)^{3/2+\varepsilon }}&\ll _\varepsilon \sum _{g\in \mathcal {U}}\left| \mathcal {V}_g\right| +\sum _{g\in \mathcal {S}_K\setminus \mathcal {U}}\left| \mathcal {V}_g\right| \\&\ll |\mathcal {U}|X+K^2\cdot \frac{X}{(\log X)^{5/2}} \end{aligned}$$

from which we infer the lower bound

$$\begin{aligned} |\mathcal {U}|\gg _\varepsilon \frac{K^2}{(\log XK)^{3/2+\varepsilon }}. \end{aligned}$$

Hence we have shown that for $\gg _\varepsilon K^2/(\log K)^{3/2+\varepsilon }$ of the forms $g\in \mathcal {S}_K$ we havewhich is what we wanted to prove. $\square$

## Proof of Theorem 1.3

### Proof of Proposition 2.1

First we explain how to use Lemmas 2.2 and 2.3 to deduce Proposition 2.1. Recall the definition of the Iwaniec–Sarnak mollifier $\mathcal {M}_{f,d}$ from Sect. 2. Let $\theta >0$ be arbitrarily small, but fixed. Also, let *h* be a compactly supported smooth function specified later. Note that by (2.4) the constraint $\sqrt{\alpha _g}|c_g(|d|)|> k^{-1/2-\theta }$ is equivalent to $\omega _f L(1/2,f\otimes \chi _d)> k^{-1-2\theta }$. Using the Cauchy–Schwarz inequality we have

$$\begin{aligned}&\left( \sum _{k\in \mathbb {Z}}h\left( \frac{2k}{K}\right) \sum _{\begin{array}{c} f\in \mathcal {B}_k\\ \omega _f L(1/2,f\otimes \chi _d)> k^{-1-2\theta } \end{array}} \omega _f L\left( \frac{1}{2},f\otimes \chi _{d}\right) \mathcal {M}_{f,d}\right) ^2\\&\quad \le \left( \sum _{k\in \mathbb {Z}}h\left( \frac{2k}{K}\right) \sum _{\begin{array}{c} f\in \mathcal {B}_k\\ \omega _f L(1/2,f\otimes \chi _d)> k^{-1-2\theta } \end{array}} \omega _f\right) \\&\quad \times \left( \sum _{k\in \mathbb {Z}}h\left( \frac{2k}{K}\right) \sum _{\begin{array}{c} f\in \mathcal {B}_k\\ \omega _f L(1/2,f\otimes \chi _d)> k^{-1-2\theta } \end{array}}\omega _f L\left( \frac{1}{2},f\otimes \chi _{d}\right) ^2\mathcal {M}_{f,d}^2 \right) . \end{aligned}$$

First we note that

$$\begin{aligned}&\sum _{k\in \mathbb {Z}}h\left( \frac{2k}{K}\right) \sum _{\begin{array}{c} f\in \mathcal {B}_k\\ \omega _f L(1/2,f\otimes \chi _d)> k^{-1-2\theta } \end{array}} \omega _f L\left( \frac{1}{2},f\otimes \chi _{d}\right) \mathcal {M}_{f,d}\\&\quad =\sum _{k\in \mathbb {Z}}h\left( \frac{2k}{K}\right) \sum _{f\in \mathcal {B}_k} \omega _f L\left( \frac{1}{2},f\otimes \chi _{d}\right) \mathcal {M}_{f,d}\\&\qquad +O\left( \sum _{k\in \mathbb {Z}}h\left( \frac{2k}{K}\right) \sum _{\begin{array}{c} f\in \mathcal {B}_k\\ \omega _f L(1/2,f\otimes \chi _d)\le k^{-1-2\theta } \end{array}} \omega _f L\left( \frac{1}{2},f\otimes \chi _{d}\right) \mathcal {M}_{f,d}\right) \\&\quad =\sum _{k\in \mathbb {Z}}h\left( \frac{2k}{K}\right) \sum _{f\in \mathcal {B}_k} \omega _f L\left( \frac{1}{2},f\otimes \chi _{d}\right) \mathcal {M}_{f,d}\\&\qquad +O\left( K^{-1-2\theta }\sum _{k\in \mathbb {Z}}h\left( \frac{2k}{K}\right) \sum _{f\in \mathcal {B}_k}|\mathcal {M}_{f,d}|\right) \\&\quad =\sum _{k\in \mathbb {Z}}h\left( \frac{2k}{K}\right) \sum _{f\in \mathcal {B}_k} \omega _f L\left( \frac{1}{2},f\otimes \chi _{d}\right) \mathcal {M}_{f,d}+O_\varepsilon \left( K^{1-2\theta +\varepsilon }\right) \\&\quad \sim \frac{K}{2}\int \limits _0^\infty h(t)\,\mathrm d t, \end{aligned}$$

where in the last step Lemma 2.2 was used and the penultimate estimate follows from the easy bound^16^

$$ \sum _{k\in \mathbb {Z}}h\left( \frac{2k}{K}\right) \sum _{f\in \mathcal {B}_k}|\mathcal {M}_{f,d}|\ll _\varepsilon K^{2+\varepsilon }, $$

which is a direct consequence of the penultimate estimate in [20, p. 164] and the Cauchy–Schwarz inequality.

On the other hand, by Lemma 2.3 and using non-negativity of the summands we have

$$\begin{aligned}&\sum _{k\in \mathbb {Z}}h\left( \frac{2k}{K}\right) \sum _{\begin{array}{c} f\in \mathcal {B}_k\\ \omega _f L(1/2,f\otimes \chi _d)> k^{-1-2\theta } \end{array}}\omega _f L\left( \frac{1}{2},f\otimes \chi _{d}\right) ^2\mathcal {M}_{f,d}^2\\&\quad \le \sum _{k\in \mathbb {Z}}h\left( \frac{2k}{K}\right) \sum _{f\in \mathcal {B}_k}\omega _f L\left( \frac{1}{2},f\otimes \chi _{d}\right) ^2\mathcal {M}_{f,d}^2\\&\quad \sim K\left( \int \limits _0^\infty h(t)\,\mathrm d t\right) \left( 1+\frac{\log |d|K}{\log L} \right) . \end{aligned}$$

Thus, by choosing the length *L* of the mollifier to be as long as possible i.e. $L=|d|^{-1}K(\log K)^{-20}$,

$$\begin{aligned}&\sum _{k\in \mathbb {Z}}h\left( \frac{2k}{K}\right) \sum _{\begin{array}{c} f\in \mathcal {B}_k\\ \omega _f L(1/2,f\otimes \chi _d)> k^{-1-2\theta } \end{array}}\omega _f\\&\quad \ge \frac{K^2}{4K}\cdot \frac{1}{1+\frac{\log |d|K}{\log L}}\left( \int \limits _0^\infty h(t)\,\mathrm d t\right) +o(K) \nonumber \\&\quad \ge \frac{K}{4}\left( 1-\frac{\log |d|}{2\log K-20\log \log K}-\frac{\log K}{2\log K-20\log \log K}+o_{K\rightarrow \infty }(1)\right) \\&\quad \times \left( \int \limits _0^\infty h(t)\,\mathrm d t\right) \nonumber \\&\quad >\frac{K}{8}\left( 1-\frac{\log |d|}{\log K}+o_{K\rightarrow \infty }(1)\right) \left( \int \limits _0^\infty h(t)\,\mathrm d t\right) \end{aligned}$$

for all fundamental discriminants $|d|\le K^c$ (with some $c>0$). As

$$ \sum _{k\in \mathbb {Z}}h\left( \frac{2k}{K}\right) \sum _{f\in \mathcal {B}_k}\omega _f\sim \frac{K}{2}\int \limits _0^\infty h(t)\,dt $$

this in particular implies

$$ \sum _{k\in \mathbb {Z}}h\left( \frac{2k}{K}\right) \sum _{\begin{array}{c} f\in \mathcal {B}_k\\ \omega _f L(1/2,f\otimes \chi _d)> {k}^{-1-2\theta } \end{array}}\omega _f>\left( \frac{1}{4}-\frac{\varepsilon }{2}\right) \sum _{k\in \mathbb {Z}}h\left( \frac{2k}{K}\right) \sum _{f\in \mathcal B_k}\omega _f $$

for large enough *K*.

Now choose the weight function *h* so that it is supported in the interval [2, 4] and is identically one in $[2+\nu ,4-\nu ]$, where $\nu >0$ is fixed and sufficiently small in terms of $\varepsilon >0$. The harmonic weights $\omega _f$ may be removed again by using [2, Lemma 8.9.] (similarly as in [20], in particular see the comments on p. 165) to get the same proportion of the event $\omega _fL(1/2,f\otimes \chi _d)>k^{-1-2\theta }$ for the natural average:

9.1 $$\begin{aligned} \sum _{k\sim K}\sum _{\begin{array}{c} f\in \mathcal {B}_k\\ \omega _f L(1/2,f\otimes \chi _d)> k^{-1-2\theta } \end{array}}1&\ge \sum _{k\in \mathbb {Z}}h\left( \frac{2k}{K}\right) \sum _{\begin{array}{c} f\in \mathcal {B}_k \\ \omega _f L(1/2,f\otimes \chi _d)> k^{-1-2\theta } \end{array}}1 \nonumber \\&>\left( \frac{1}{4}-\frac{\varepsilon }{2}\right) \sum _{k\in \mathbb {Z}}h\left( \frac{2k}{K}\right) \sum _{f\in \mathcal {B}_k}1 \nonumber \\&\ge \left( \frac{1}{4}-\frac{\varepsilon }{2}\right) (1-\nu )\#\mathcal {S}_K \nonumber \\&\ge \left( \frac{1}{4}-\varepsilon \right) \#\mathcal {S}_K. \end{aligned}$$

for large enough *K*. This completes the proof of Proposition 2.1.

### Sign changes of Fourier coefficients along squares

The final thing to do is to use Proposition 2.1 to complete the proof of Theorem 1.3. Recall that our aim is to produce many pairs of odd integers for which (8.1) holds. For this we seek such pairs in short intervals and the idea here is to connect short sums of the Fourier coefficients to long sums using the results of [39]. Let $\eta >0$ be as in Proposition 5.1. Given $g\in \mathcal {S}_K$, fix any odd fundamental discriminant *d* and define the multiplicative function $h_g:\mathbb {N}\longrightarrow \{-1,0,1\}$ by setting

$$ h_g(p^\ell ):={\left\{ \begin{array}{ll} \text {sgn}(c_g(|d|)^{-1}c_g(|d|p^{2\ell })) & \quad \text {if }|c_g(|d|)^{-1}c_g(|d|p^{2\ell })|\ge p^{-\ell \eta /2}\,\text {and }p>2\\ 0 & \quad \text {otherwise} \end{array}\right. } $$

Here $\text {sgn}(x)$ is the sign of $x\in \mathbb {R}$ and we interpret $c_g(|d|)^{-1}=0$ if $c_g(|d|)=0$.

Note that if $h_g(m)\ne 0$, then *m* is odd, $c_g(|d|)\ne 0$, $|c_g(|d|m^2)|\ge |c_g(|d|)|m^{-\eta /2}$, and finally $h_g(m)=\text {sgn}(c_g(|d|)^{-1}c_g(|d|m^2))$. These follow from the fact that $m\mapsto c_g(|d|m^2)$ satisfy the multiplicative property $c_g(|d|)c_g(|d|m^2n^2)=c_g(|d|m^2)c_g(|d|n^2)$ (when $(m,n)=1$) [49, (1.18)] for any fixed fundamental discriminant *d*. From this it follows that the map $m\mapsto c_g(|d|)^{-1}c_g(|d|m^2)$ defines a multiplicative function when $c_g(|d|)\ne 0$.

Let $\theta >0$ be a fixed quantity chosen later and define

$$ S_{K,d}:=\bigcup _{k\sim K}\{g\in B_{k+\frac{1}{2}}^+:\,\sqrt{\alpha _g}|c_g(|d|)|> k^{-1/2-\theta }\}. $$

To prove Theorem 1.3 we adapt the argument of [32, Section 3]. We start with a key auxiliary result.

#### Lemma 9.1

Let $\varepsilon >0$ be arbitrary but fixed, and $d\ll 1$ be a fixed fundamental discriminant.^17^ Then there exists $X_0(\varepsilon )$ such that for $X_0<X<K$ we have, for all but at most $\varepsilon \# S_{K,d}$ forms $g\in S_{K,d}$,

$$ \left| \frac{1}{X}\sum _{n\sim X}|h_g(n)|-\frac{1}{X}\left| \sum _{n\sim X}h_g(n)\right| \right| \gg _{\eta ,\varepsilon } 1. $$

#### Proof

It suffices to show the following two assertions:

1. For all but at most $(\varepsilon /2)\#S_{K,d}$ of the forms $g\in S_{K,d}$ we have
  $$ \frac{1}{X}\sum _{n\sim X}|h_g(n)|\gg _{\eta ,\varepsilon }1,$$
  where the implicit constant is independent of *g*.
2. For all but at most $(\varepsilon /2)\#S_{K,d}$ of the forms $g\in S_{K,d}$ we have
  $$ \frac{1}{X}\sum _{n\sim X}h_g(n)=o(1). $$

Towards (1) we argue as follows. Let us first assume that the fixed fundamental discriminant *d* is positive. Then note that by the identity (2.1), the triangle inequality, and Lemma 4.6 we have

$$\begin{aligned}&\sum _{\begin{array}{c} k\sim K\\ k\equiv 0\,(2) \end{array}}\sum _{\begin{array}{c} g\in B_{k+\frac{1}{2}}^+\\ \sqrt{\alpha _g}|c_g(|d|)|>k^{-1/2-\theta } \end{array}}\sum _{\begin{array}{c} p\le X\\ |c_g(|d|)^{-1}c_g(|d|p^2)|<p^{-\eta /2} \end{array}}\frac{1}{p} \\&\ll \sum _{\begin{array}{c} k\sim K\\ k\equiv 0\,(2) \end{array}}\sum _{f\in \mathcal {B}_k}\sum _{\begin{array}{c} p\le X\\ |\lambda _f(p)-\chi _d(p)/\sqrt{p}|<p^{-\eta /2} \end{array}}\frac{1}{p} \\&\le \sum _{\begin{array}{c} k\sim K\\ k\equiv 0\,(2) \end{array}}\sum _{f\in \mathcal {B}_k}\sum _{\begin{array}{c} p\le X\\ |\lambda _f(p)|<p^{-\eta /2}+1/\sqrt{p} \end{array}}\frac{1}{p} \\&\ll \sum _{\begin{array}{c} k\sim K\\ k\equiv 0\,(2) \end{array}} \#\mathcal {B}_k\sum _{p\le X}\left( p^{-3/2}+p^{-1-\eta /2}+\frac{\log p}{p\log k}\right) \\&\ll _\eta \sum _{\begin{array}{c} k\sim K\\ k\equiv 0\,(2) \end{array}}\#\mathcal {B}_k\\&\ll \sum _{\begin{array}{c} k\sim K\\ k\equiv 0\,(2) \end{array}}\sum _{\begin{array}{c} g\in B_{k+\frac{1}{2}}^+\\ \sqrt{\alpha _g}|c_g(|d|)|>k^{-1/2-\theta } \end{array}}1, \end{aligned}$$

where in the last step we have used (9.1) (together with (2.4) and the fact that $c_g(|d|)=0$ for positive *d* when *k* is odd), and the fact that $\#\mathcal {B}_k\asymp k$.

Hence there is a positive constant $C_1$ depending only at most on $\eta$ and $\theta$ such that for any given $\varepsilon >0$,

$$\begin{aligned} \sum _{\begin{array}{c} p\le X \\ h_g(p)=0 \end{array}}\frac{1}{p}\le \frac{C_1}{\varepsilon } \end{aligned}$$

for all but at most $(\varepsilon /2)\# I_{K,d}$ forms $g\in I_{K,d}$, where

$$\begin{aligned} I_{K,d}:=\bigcup _{\begin{array}{c} k\sim K\\ k\equiv 0\,(2) \end{array}}\{g\in B_{k+\frac{1}{2}}^+{:}\,\sqrt{\alpha _g}|c_g(|d|)|>k^{-1/2-\theta }\}. \end{aligned}$$

Similarly, for a fixed negative fundamental discriminant *d*, one shows that there is a positive constant $C_2$ depending only at most on $\eta$ and $\theta$ such that for any given $\varepsilon >0$,

$$\begin{aligned} \sum _{\begin{array}{c} p\le X \\ h_g(p)=0 \end{array}}\frac{1}{p}\le \frac{C_2}{\varepsilon } \end{aligned}$$

for all but at most $(\varepsilon /2)\# J_{K,d}$ forms $g\in J_{K,d}$, where

$$\begin{aligned} J_{K,d}:=\bigcup _{\begin{array}{c} k\sim K\\ k\equiv 1\,(2) \end{array}}\{g\in B_{k+\frac{1}{2}}^+{:}\,\sqrt{\alpha _g}|c_g(|d|)|>k^{-1/2-\theta }\}. \end{aligned}$$

Note that for any given *d*, $S_{K,d}=I_{K,d}\cup J_{K,d}$ with one of the sets $I_{K,d},\, J_{K,d}$ being empty (as $c_g(|d|)=0$ when $(-1)^kd<0)$. Now the first assertion follows from [15, Theorem 2].

For the second assertion, observe that, for a fixed positive fundamental discriminant *d*,

9.2 $$\begin{aligned} \sum _{\begin{array}{c} p\le X\\ h_g(p)=-1 \end{array}}\frac{1}{p}=\sum _{\begin{array}{c} p\le X\\ c_g(|d|)^{-1}c_g(|d|p^2)<0 \end{array}}\frac{1}{p}-\sum _{\begin{array}{c} p\le X\\ -p^{-\eta /2}<c_g(|d|)^{-1}c_g(|d|p^2)<0 \end{array}}\frac{1}{p}. \end{aligned}$$

Recalling (2.1) and using the fact that $\chi _d(p)\in \{-1,0,1\}$ it follows that the first sum on the right-hand side of (9.2) is

$$\begin{aligned}&\ge \sum _{\begin{array}{c} p\le X\\ \lambda _f(p)<-1/\sqrt{p} \end{array}}\frac{1}{p} \\&\ge \sum _{\begin{array}{c} p\le X\\ \lambda _f(p)<0 \end{array}}\frac{1}{p}-\sum _{\begin{array}{c} p\le X\\ -1/\sqrt{p}<\lambda _f(p)<0 \end{array}}\frac{1}{p}. \end{aligned}$$

Arguing identically as in [32, p. 1610] we have that

$$\begin{aligned} \sum _{\begin{array}{c} p\le X\\ \lambda _f(p)<0 \end{array}}\frac{1}{p}\ge \frac{1+o(1)}{8}\log \log X+\sum _{\log X\le p\le X^{1/1000}}\frac{\lambda _f(p^2)-2\lambda _f(p)}{p} \end{aligned}$$

and using the large sieve inequality of Lemma 4.5 the latter sum on the right-hand side contributes $o(\log \log X)$ for almost all forms $f\in \cup _{k\sim K\,\text {even}}\mathcal {B}_k$.

To summarise, we have deduced that

$$\begin{aligned} \sum _{\begin{array}{c} p\le X\\ h_g(p)=-1 \end{array}}\frac{1}{p}\ge \frac{(1+o(1))}{8}\log \log X-\sum _{\begin{array}{c} p\le X\\ -1/\sqrt{p}<\lambda _f(p)<0 \end{array}}\frac{1}{p}-\sum _{\begin{array}{c} p\le X\\ -p^{-\eta /2}<c_g(|d|)^{-1}c_g(|d|p^2)<0 \end{array}}\frac{1}{p} \end{aligned}$$

for almost all forms $g\in \cup _{k\sim K\,\text {even}}B_{k+1/2}^+$. But from the arguments used to establish the first assertion above we know that there is an absolute constant $C_3$ so that

$$ \sum _{\begin{array}{c} p\le X\\ -p^{-\eta /2}<c_g(|d|)^{-1}c_g(|d|p^2)<0 \end{array}}\frac{1}{p}\le \frac{C_3}{\varepsilon } $$

for all but $(\varepsilon /4)\# I_{K,d}$ of the forms $g\in I_{K,d}$. A similar computation also shows that there exists an absolute constant $C_4$ so that

$$ \sum _{\begin{array}{c} p\le X\\ -1/\sqrt{p}<\lambda _f(p)<0 \end{array}}\frac{1}{p}\le \frac{C_4}{\varepsilon } $$

for all but $(\varepsilon /4)\# I_{K,d}$ of the forms $g\in I_{K,d}$ (recall here that $f\in \mathcal {B}_k$ corresponds to some $g\in B_{k+\frac{1}{2}}^+$ under the Shimura correspondence). Thus for all but at most $(\varepsilon /2)\#I_{K,d}$ of the forms $g\in I_{K,d}$ we have

$$ \sum _{\begin{array}{c} p\le X\\ h_g(p)=-1 \end{array}}\frac{1}{p}\ge \frac{(1+o(1))}{8}\log \log X. $$

Similarly one shows that the same estimate holds for all but $(\varepsilon /2)\# J_{K,d}$ of the forms $g\in J_{K,d}$ when the fixed fundamental discriminant *d* is negative. This finishes the proof of (2) by Halász’s theorem (Lemma 4.9) by recalling that one of the sets $I_{K,d},\,J_{K,d}$ is empty for any given *d*. This concludes the proof of the lemma. $\square$

To finish the proof of Theorem 1.3 we argue as follows. Set now $\theta =\eta /4$, and let $\varepsilon >0$ be arbitrarily small, but fixed. Note that by definition, with $|d|\ll 1$ being fixed and choosing $X\asymp \sqrt{K/|d|Y}$, a sign change of $h_g$ on [*X*, 2*X*] means the existence of odd natural numbers *m* and *n* at most 2*X* so that

$$ c_g(|d|m^2)\le -|c_g(|d|)|m^{-\eta /2}<|c_g(|d|)|n^{-\eta /2}\le c_g(|d|n^2). $$

Multiplying both sides by $\sqrt{\alpha _g}$ we require

$$ \sqrt{\alpha _g}c_g(|d|m^2)\le -\sqrt{\alpha _g}|c_g(|d|)|m^{-\eta /2}<\sqrt{\alpha _g}|c_g(|d|)|n^{-\eta /2}\le \sqrt{\alpha _g}c_g(|d|n^2). $$

But if $\sqrt{\alpha _g}|c_g(|d|)|> k^{-1/2-\eta /4}$ (which holds for at least $(1/4-\varepsilon /4)\#\mathcal {S}_K$ of the forms $g\in \mathcal {S}_K$ by Proposition 2.1) this implies, as e.g. $n\le 2X\ll \sqrt{k}$,

$$ \sqrt{\alpha _g}c_g(|d|m^2)<-k^{-\delta }<k^{-\delta }<\sqrt{\alpha _g}c_g(|d|n^2), $$

where $\delta =1/2+\eta /2$. As discussed in the beginning of Sect 8, this guarantees an existence of a zero on both of the lines $\delta _1$ and $\delta _2$.

Thus it suffices to exhibit sign changes of the multiplicative function $h_g$. Let us first fix a positive odd fundamental discriminant with $d_1\ll 1$ (one can e.g. take $d_1=5$) and denote the corresponding function $h_g$ by $h_g^{(1)}$. Suppose $H=H(\eta ,\varepsilon )$ is sufficiently large fixed constant and $X_0(\eta ,\varepsilon )<X\asymp \sqrt{K/|d_1|Y}$. By Proposition 2.1 we know that $\sqrt{\alpha _g}|c_g(|d_1|)|>k^{-1/2-\eta /4}$ for at least $(1/4-\varepsilon /4)\#\mathcal {S}_K$ of the forms $g\in \mathcal {S}_K$. Then using Lemmas 4.8 and 9.1 we have that for at least $(1-\varepsilon /4)\cdot (1/4-\varepsilon /4)\#\mathcal {S}_K\ge (1/4-\varepsilon /2)\#\mathcal {S}_K$ of the forms $g\in \mathcal {S}_K$,

$$\begin{aligned}&\frac{1}{H}\sum _{x\le n\le x+H}|h_g^{(1)}(n)|-\frac{1}{H}\left| \sum _{x\le n\le x+H}h_g^{(1)}(n)\right| \\&\quad =\frac{1}{X}\sum _{n\sim X}|h_g^{(1)}(n)|-\frac{1}{X}\left| \sum _{n\sim X}h_g^{(1)}(n)\right| +O\left( (\log H)^{-1/200}\right) \\&\quad \gg _{\eta ,\varepsilon }1 \end{aligned}$$

for all $x\sim X$ outside an exceptional set of size at most $CX(\log H)^{-1/100}$ for some absolute constant $C>1$. This implies that for each such form $g\in S_K$ there exist numbers $X\le x_1<\cdots <x_N\le 2X$ with $x_{j+1}-x_j>H$ and $N\ge \frac{1}{10}\cdot \frac{X}{H}$ such that

$$ \left| \frac{1}{H}\sum _{x_j\le n\le x_j+H}|h_g^{(1)}(n)|-\frac{1}{H}\left| \sum _{x_j\le n\le x_j+H}h_g^{(1)}(n)\right| \right| \gg 1 $$

for each $j=1,...,N$. We conclude that each interval $[x_j,x_j+H]$ yields a sign change of $h_g^{(1)}(n)$ for at least $(1/4-\varepsilon /2)\#\mathcal {S}_K$ of the forms $g\in \mathcal {S}_K$ above. As discussed in the introduction, this leads to

$$ \gg \frac{X}{H}\gg \sqrt{\frac{K}{Y}} $$

zeroes on both of the lines $\delta _1$ and $\delta _2$ with $\text {Im}(z)\ge Y$ for at least $(1/4-\varepsilon /2)\#\mathcal {S}_K$ of the forms $g\in \mathcal {S}_K$.

A similar argument shows that fixing a negative odd fundamental discriminant with $d_2$ with $|d_2|\ll 1$ (e.g. $d_2=-3$), the corresponding function $h_g^{(2)}$ has $\gg \sqrt{K/Y}$ sign changes for at least $(1/4-\varepsilon /2)\#\mathcal {S}_K$ of the forms $g\in \mathcal {S}_K$. But as at most one of the inequalities $\sqrt{\alpha _g}|c_g(|d_1|)|>k^{-1/2-\eta /4}$ and $\sqrt{\alpha _g}|c_g(|d_2|)|>k^{-1/2-\eta /4}$ can hold for a given $g\in B_{k+\frac{1}{2}}^+$ (as $d_1d_2<0$ and $c_g(|d|)=0$ whenever $(-1)^k d<0$) it follows that the subsets of $\mathcal {S}_K$ for which $h_g^{(1)}$ and $h_g^{(2)}$ exhibit sign changes are disjoint. Thus we get $\gg \sqrt{K/Y}$ real zeroes on both of the line segments $\delta _1$ and $\delta _2$ for at least $(1/2-\varepsilon )\#\mathcal {S}_K$ forms in $\mathcal {S}_K$. This completes the proof. $\square$

## Data Availability

No data was gathered for this work.

## Footnotes

1 In the same paper they also considered zeroes on the arc $\{|z|=1\}$ where $z^{k/2}f(z)$ is real-valued.

2 Here *B*(*x*, *r*) denotes the circle with radius *r* centred at *x*.

3 Indeed, the numbers $c_g(n)n^{k/2-1/4}$ lie in the field generated over $\mathbb Q$ by the Fourier coefficients of its Shimura lift, which is a level 1 Hecke eigenform of weight 2*k*, so these numbers are real and algebraic (see [28, Proposition 4.2] and also the remarks before [25, Theorem 1]).

4 Here, and throughout the paper, the notation $\ell \sim L$ means that $\ell \in [L,2L]\cap \mathbb N$. This should not be confused with the asymptotic notation $f\sim g$ for functions *f* and *g* used also in the present article.

5 However, for elements in certain canonical basis, see [11].

6 In the integral weight case a probabilistic model for this is given by Ghosh and Sarnak [12, Section 6] and the same model works also in the half-integral weight setting.

7 One may refine this conjecture to state that each of the individual line segments $\delta _j\cap \mathcal {F}_Y$ contain $50\%$ of the zeroes.

8 In reality we need something a bit stronger, but for the purposes of sketching the argument we pretend here that it suffices to find sign changes.

9 Notice that the leading constants in the main terms in [20, Theorem 3] should be halved.

10 In our notation the length *L* of the mollifier corresponds to the parameter *M* in [20].

11 Sometimes we write $\#\mathcal {X}$ for the cardinality of a set $\mathcal {X}$.

12 When $j=4$ an upper bound of the right order of magnitude suffices for our purposes, but here we focus on the asymptotic evaluation as this would be needed for the incorporation of a mollifier to get a result that holds for a positive proportion of forms.

13 Note that in general the estimate in [9] only holds for squarefree *m*. However, in our case *g* is a Hecke eigenform and in this case the estimate can be extended for arbitrary $m\in \mathbb N$, see [21, Lemma 3.3.].

14 Note that in [53] the conductor of the quadratic character was assumed to be odd, but the same result holds for even discriminants as well [1, Appendix A].

15 Here $\mathbb A$ denotes the adele ring of rational numbers.

16 Note that the normalisation in [20] differs slightly from ours.

17 Recall that the function $h_g$ depends on *d*.

## References

[1] Andersen, N., Duke, W.D.: Modular invariants for real quadratic fields and Kloosterman sums. Algebra Number Theory **14**(6), 1537–1575 (2020)

[2] Balkanova, O., Frolenkov, D.: Moments of -functions and the Liouville–Green method. J. Eur. Math. Soc. (JEMS) **23**(4), 1333–1380 (2021)

[3] Blomer, V., Harcos, G., Michel, P.: A Burgess-like subconvex bound for twisted -functions. Forum Math. **19**(1), 61–105 (2007). Appendix 2 by Z. Mao

[4] Blomer, V.: Non-vanishing of class group -functions at the central point. Ann. Inst. Fourier (Grenoble) **54**(4), 831–847 (2004)

[5] Blomer, V.: On the central value of symmetric square -functions. Math. Z. **260**(4), 755–777 (2008)

[6] Blomer, V., Corbett, A.: A symplectic restriction problem. Math. Ann. **382**(3–4), 1323–1424 (2022)

[7] Blomer, V., Harcos, G.: Hybrid bounds for twisted -functions. J. Reine Angew. Math. **621**, 53–79 (2008)

[8] Bruinier, J.H., Kohnen, W.: Sign changes of coefficients of half integral weight modular forms. In: Modular Forms on Schiermonnikoog, pp. 57–65. Cambridge University Press, Cambridge (2008)

[9] Conrey, J.B., Iwaniec, H.: The cubic moment of central values of automorphic -functions. Ann. Math. (2) **151**(3), 1175–1216 (2000)

[10] Deshouillers, J.-M., Iwaniec, H.: Kloosterman sums and Fourier coefficients of cusp forms. Invent. Math. **70**(2), 219–288 (1982)

[11] Folsom, A., Jenkins, P.: Zeros of modular forms of half integral weight. Res. Number Theory **2**, 23 (2016)

[12] Ghosh, A., Sarnak, P.: Real zeros of holomorphic Hecke cusp forms. J. Eur. Math. Soc. (JEMS) **14**(2), 465–487 (2012)

[13] Hall, R.R., Tenenbaum, G.: Effective mean value estimates for complex multiplicative functions. Math. Proc. Camb. Philos. Soc. **110**(2), 337–351 (1991)

[14] Harper, A.: Sharp Conditional Bounds for Moments of the Riemann Zeta Function. arXiv:1305.4618 (2013)

[15] Hildebrand, A.: Quantitative mean value theorems for nonnegative multiplicative functions. II. Acta Arith. **48**(3), 209–260 (1987)

[16] Holowinsky, R., Soundararajan, K.: Mass equidistribution for Hecke eigenforms. Ann. Math. (2) **172**(2), 1517–1528 (2010)

[17] Hulse, T.A., Kiral, E.M., Kuan, C.I., Lim, L.-M.: The sign of Fourier coefficients of half-integral weight cusp forms. Int. J. Number Theory **8**(3), 749–762 (2012)

[18] Iwaniec, H., Kowalski, E.: Analytic Number Theory. American Mathematical Society Colloquium Publications, vol. 53. American Mathematical Society, Providence (2004)

[19] Iwaniec, H., Luo, W., Sarnak, P.: Low lying zeros of families of -functions. Inst. Hautes Études Sci. Publ. Math. **91**, 55–131 (2000)

[20] Iwaniec, H., Sarnak, P.: The non-vanishing of central values of automorphic -functions and Landau-Siegel zeros. Isr. J. Math. **120**, 155–177 (2000)

[21] Jiang, Y.-J., Lau, Y.-K., Lü, G.-S., Royer, E., Wu, J.: On Fourier coefficients of modular forms of half integral weight at squarefree integers. Math. Z. **293**(1–2), 789–808 (2019)

[22] Khan, R.: Non-vanishing of the symmetric square -function at the central point. Proc. Lond. Math. Soc. (3) **100**(3), 736–762 (2010)

[23] Knopp, M., Kohnen, W., Pribitkin, W.: On the Signs of Fourier Coefficients of Cusp Forms. vol. 7, pp. 269–277. Rankin Memorial Issues (2003)

[24] Kohnen, W., Lau, Y.-K., Wu, J.: Fourier coefficients of cusp forms of half-integral weight. Math. Z. **273**(1–2), 29–41 (2013)

[25] Kohnen, W., Zagier, D.: Values of -series of modular forms at the center of the critical strip. Invent. Math. **64**(2), 175–198 (1981)

[26] Kohnen, W.: Newforms of half-integral weight. J. Reine Angew. Math. **333**, 32–72 (1982)

[27] Kowalski, E., Michel, P.: The analytic rank of and zeros of automorphic -functions. Duke Math. J. **100**(3), 503–542 (1999)

[28] Kumar, N., Purkait, S.: A note on the Fourier coefficients of half-integral weight modular forms. Arch. Math. (Basel) **102**(4), 369–378 (2014)

[29] Lau, Y.-K., Wu, J.: A large sieve inequality of Elliott–Montgomery–Vaughan type for automorphic forms and two applications. Int. Math. Res. Not. IMRN. (5) **2008**, rnm162 (2008)

[30] Lau, Y., Royer, E., Jie, W.: Sign of Fourier coefficients of modular forms of half-integral weight. Mathematika **62**(3), 866–883 (2016)

[31] Lau, Y.-K., Jie, W.: A density theorem on automorphic -functions and some applications. Trans. Am. Math. Soc. **358**(1), 441–472 (2006)

[32] Lester, S., Matomäki, K., Radziwiłł, M.: Small scale distribution of zeros and mass of modular forms. J. Eur. Math. Soc. (JEMS) **20**(7), 1595–1627 (2018)

[33] Lester, S., Radziwiłł, M.: Quantum unique ergodicity for half-integral weight automorphic forms. Duke Math. J. **169**(2), 279–351 (2020)

[34] Lester, S., Radziwiłł, M.: Signs of Fourier coefficients of half-integral weight modular forms. Math. Ann. **379**(3–4), 1553–1604 (2021)

[35] Li, X.: Moments of quadratic twists of modular -functions. Invent. Math. **237**(2), 697–733 (2024)

[36] Luo, W., Sarnak, P.: Mass equidistribution for Hecke eigenforms. Commun. Pure Appl. Math. **56**(7), 874–891 (2003). Dedicated to the memory of Jürgen K. Moser

[37] Matomäki, K.: Real zeros of holomorphic Hecke cusp forms and sieving short intervals. J. Eur. Math. Soc. (JEMS) **18**(1), 123–146 (2016)

[38] Matomäki, K., Radziwiłł, M.: Sign changes of Hecke eigenvalues. Geom. Funct. Anal. **25**(6), 1937–1955 (2015)

[39] Matomäki, K., Radziwiłł, M.: Multiplicative functions in short intervals. Ann. Math. (2) **183**(3), 1015–1056 (2016)

[40] Murty, M.R., Sinha, K.: Effective equidistribution of eigenvalues of Hecke operators. J. Number Theory **129**(3), 681–714 (2009)

[41] Natalini, P., Palumbo, B.: Inequalities for the incomplete gamma function. Math. Inequal. Appl. **3**(1), 69–77 (2000)

[42] Nelson, P.D.: Equidistribution of cusp forms in the level aspect. Duke Math. J. **160**(3), 467–501 (2011)

[43] Petrow, I., Young, M.P.: A generalized cubic moment and the Petersson formula for newforms. Math. Ann. **373**(1–2), 287–353 (2019)

[44] Radziwiłł, M., Soundararajan, K.: Moments and distribution of central -values of quadratic twists of elliptic curves. Invent. Math. **202**(3), 1029–1068 (2015)

[45] Rankin, F.K.C., Swinnerton-Dyer, H.P.F.: On the zeros of Eisenstein series. Bull. Lond. Math. Soc. **2**, 169–170 (1970)

[46] Rudnick, Z.: On the asymptotic distribution of zeros of modular forms. Int. Math. Res. Not. **34**, 2059–2074 (2005)

[47] Rudnick, Z., Sarnak, P.: The behaviour of eigenstates of arithmetic hyperbolic manifolds. Commun. Math. Phys. **161**(1), 195–213 (1994)

[48] Sarnak, P.: Estimates for Rankin–Selberg -functions and quantum unique ergodicity. J. Funct. Anal. **184**(2), 419–453 (2001)

[49] Shimura, G.: On modular forms of half integral weight. Ann. Math. **2**(97), 440–481 (1973)

[50] Soundararajan, K., Young, M.P.: The second moment of quadratic twists of modular -functions. J. Eur. Math. Soc. (JEMS) **12**(5), 1097–1116 (2010)

[51] Soundararajan, K.: Moments of the Riemann zeta function. Ann. Math. (2) **170**(2), 981–993 (2009)

[52] Waibel, F.: Fourier coefficients of half-integral weight cusp forms and Waring’s problem. Ramanujan J. **47**(1), 185–200 (2018)

[53] Young, M.P.: Weyl-type hybrid subconvexity bounds for twisted -functions and Heegner points on shrinking sets. J. Eur. Math. Soc. (JEMS) **19**(5), 1545–1576 (2017)

